# Biopolymer-based seed biopriming with fungal endophytes suppresses Fusarium wilt and modulates growth in tomato

**DOI:** 10.3389/fpls.2026.1931116

**Published:** 2026-09-11

**Authors:** Victoria Huertas, Melanie D. Gómez-Herrera, Fernando Diánez, Mila Santos

**Affiliations:** 1 Departamento de Agronomía, Escuela Superior de Ingeniería, Universidad de Almería, Almería, Spain; 2 Instituto de Química Básica y Aplicada del Nordeste Argentino, IQUIBA-NEA, UNNE-CONICET, Corrientes, Argentina

**Keywords:** biocontrol, biopolymer, cold storage, dark septate endophytes, endophytic fungi, *Fusarium oxysporum* f. sp. lycopersici, gum arabic, seed biopriming

## Abstract

Biopolymer-based seed biopriming is an emerging strategy for delivering endophytic fungal biocontrol agents while maintaining seed viability and fungal persistence, yet the compatibility of carrier matrices with dark septate endophytes (DSE), and the potential of this fungal group against *Fusarium oxysporum* f. sp. lycopersici (FOL) in tomato, remain unexplored. Three biopolymer matrices — sodium alginate (SA), gum arabic (GA) and guar gum (GG) — were evaluated as carriers for ten endophytic fungal isolates: *Torula* sp. (BC01, BC11), *Sordaria fimicola* (BC06), *Clarireedia calopus* (BC17, BC18), *Trichoderma gamsii* (BC20), *Clarireedia narcissi* (BC34), *T. saturnisporum* (BC49), *T. aggressivum* f. sp. *europaeum* (TA) and *T. longibrachiatum* (H1). Three imbibition times and four concentrations per matrix were compared, and their effects on seed germination and vigour, fungal propagule viability and entrapment efficiency, persistence during cold storage at 4 °C, and biocontrol efficacy against FOL were assessed relative to substrate inoculation. Imbibition for 15 min gave the highest and most consistent germination and vigour, preserving germination above 80% with SA (0.5–1% w/v) and GA (25–40% w/v). Propagule type determined biopolymer compatibility: spore-producing isolates reached maximum viability with GA at medium-to-high concentrations, whereas microsclerotia-producing isolates were better preserved with SA. BC20 combined with GA was incompatible with the crop, producing no seedling emergence across three independent sowings. Cold storage for 30 days maintained viable fungal loads, with BC49, H1 and TA showing the greatest persistence and BC06, BC20 and BC34 the most pronounced decline; initial entrapment and long-term viability were not correlated. Germination and seedling vigour index remained stable throughout 40 days. Biopriming consistently outperformed substrate inoculation, with BC34 and H1 achieving 100% and 94.1% protection, whereas substrate inoculation with BC01 gave negative protection (−29.4%). Principal component analysis separated both application methods, associating biopriming with greater disease suppression and substrate inoculation with greater biomass accumulation, revealing a trade-off between biocontrol efficacy and growth promotion. Individually optimized biopolymer–endophyte formulations therefore represent a viable strategy for sustainable Fusarium wilt management in tomato, and provide the first evidence that DSE propagules can be encapsulated and delivered by seed biopriming.

## 1 Introduction

The transition toward more sustainable agricultural systems has become a global priority in response to the intensive use of agrochemicals, excessive water consumption, and the progressive degradation of soil quality. These pressures threaten ecosystem functioning, food security, and human health (Firmanda et al., 2024; Xing et al., 2025; Song et al., 2024). In particular, the continued use of highly soluble mineral fertilizers has been linked to soil acidification, reduced microbial diversity, and poor nutrient use efficiency, leading to substantial losses through leaching, runoff, denitrification, and volatilization (Gowtham et al., 2024; Ramatsitsi and Manyevere, 2025). As a result, agricultural policies and research agendas increasingly emphasize the need to reduce dependence on conventional fertilizers and to promote inputs and technologies with lower environmental impact.

Among the most promising alternatives are slow- and controlled-release fertilizers (SRF/CRF) based on biopolymers, which can improve nutrient use efficiency while limiting diffuse pollution (Malik et al., 2022; Firmanda et al., 2024; Song et al., 2024). In these systems, nutrients are embedded in renewable polymeric matrices such as starch, alginate, chitosan, lignin, and cellulose, allowing nutrient release to be modulated by both material properties and environmental conditions (Malik et al., 2022; Verma et al., 2022). Because these materials are generally derived from natural resources, they are often biodegradable and biocompatible, offering an advantage over persistent synthetic coatings that can contribute to microplastic accumulation in agricultural soils (Fontana et al., 2021; Krasnopeeva et al., 2022).

Biopolymer-based hydrogels and superabsorbent polymers (SAPs) represent another relevant class of materials for sustainable crop production because of their exceptional water-holding capacity. These materials can absorb and retain water in amounts many times greater than their dry weight and can function both as soil conditioners and as carriers for nutrients and agrochemicals (Verma et al., 2022; Morales-Vargas et al., 2024; Djafaripetroudy et al., 2025). When incorporated into soil or applied as seed-coating materials, they can improve water availability in the rhizosphere, reduce irrigation frequency, enhance soil physicochemical properties, and act as slow-release delivery systems for fertilizers and pesticides (Verma et al., 2022; Song et al., 2025). Their use has also been associated with improved seedling establishment, crop growth, and tolerance to abiotic stresses such as drought and salinity, particularly in arid and semi-arid environments (Djafaripetroudy et al., 2025).

At the same time, endophytic fungi have emerged as key biological partners for reducing chemical inputs and enhancing crop performance under stressful conditions (Verma et al., 2022; Gowtham et al., 2024; Huertas et al., 2024). These microorganisms colonize internal plant tissues without causing disease symptoms and can beneficially modulate host physiology through multiple mechanisms. They improve the acquisition of nutrients including nitrogen (N), phosphorus (P), potassium (K), and micronutrients; stimulate osmolyte accumulation; activate antioxidant defenses; and influence plant hormone signaling. Together, these effects can enhance plant growth, photosynthetic performance, and tolerance to abiotic stresses such as drought and salinity (Yan et al., 2019; Gowtham et al., 2024; Rhouma et al., 2024; Majhi et al., 2025). In addition, many fungal endophytes act as biocontrol agents by suppressing phytopathogens through competition, antibiosis, mycoparasitism, and the induction of plant defense responses (Ahmed et al., 2020; MaChado et al., 2022; Akram et al., 2023; Fite et al., 2023). Genera such as *Trichoderma*, *Clonostachys*, *Piriformospora*, and several dark septate endophytes (DSE) have shown the capacity to reduce the incidence and severity of diseases caused by *Fusarium* spp. and other pathogens while also promoting plant growth (Kthiri et al., 2020; Jamil, 2021; Amaral et al., 2022; Dalimunthe et al., 2023; Mariani et al., 2024; Huertas et al., 2026).

Despite this potential, the direct use of endophytic fungal inoculants under field conditions remains constrained by several practical limitations, including low propagule stability, short shelf life, difficulties in storage and transport, rapid viability loss under environmental stress, and inconsistent establishment in soil and host tissues (Gowtham et al., 2024; Malik et al., 2022). To address these limitations, biopolymers in the form of hydrogels, microcapsules, and porous matrices have been proposed as delivery and encapsulation systems for bioinoculants. Such matrices can protect fungal propagules, facilitate handling and application, and enable gradual inoculum release, thereby potentially improving persistence and biological performance after application (Saberi-Riseh et al., 2021; Szopa et al., 2022; Pereira et al., 2023). Similarly, biopolymer-based nanocarriers and nanopesticide platforms have been explored as tools to regulate the release of agrochemicals and microorganisms, reduce environmental toxicity, and support more precise crop management strategies (Fontana et al., 2021; Saberi-Riseh et al., 2021; Szopa et al., 2022).

Three polysaccharide matrices were selected to span contrasting physicochemical behaviours rather than variants of a single material, since the coating polymer is itself a determinant of inoculant viability, coating efficacy and subsequent seed performance, and side-by-side comparisons of carrier materials remain scarce (Rocha et al., 2019; Chin et al., 2021; Cardarelli et al., 2022; Paravar et al., 2023). Sodium alginate was included as the anionic, gelling matrix: a linear polysaccharide of mannuronic and guluronic residues that crosslinks with divalent cations into non-toxic, biodegradable hydrogels with high water-holding capacity, and the most widely used carrier for microbial encapsulation, controlled release and seed coating in agriculture (Skrzypczak et al., 2021; Korbecka-Glinka et al., 2022; Martínez-Cano et al., 2022). In biopriming systems it has shown consistently high compatibility with both seeds and inoculants, achieving efficient entrapment of *Trichoderma asperellum* propagules on pak choi and chilli seeds and sustaining high viable loads during storage and disease control in cucumber and tobacco (Chin et al., 2021, 2022; Abdukerim et al., 2024; Jiang et al., 2025).

Gum arabic was selected as the non-gelling, adhesive counterpart: a highly branched arabinogalactan–protein complex whose amphiphilic character confers emulsifying capacity, high solubility and low viscosity even at high solid contents. It is a well-established binder in microbial seed coating, improving the affinity between the seed coat and the coating layer, protecting inoculants against desiccation and extending microbial survival on the seed (Rocha et al., 2019; Monisha et al., 2023; Paravar et al., 2023). Reports that excessive gum arabic films can delay early germination in cucumber indicate that its performance is strongly dependent on concentration and film properties (Skrzypczak et al., 2021; Paravar et al., 2023). Guar gum, a neutral galactomannan that develops high viscosity without gelling, was included as a low-cost sticking agent allowing viscosity effects to be separated from ionic gelation and matrix charge; although less documented than the other two, it has supported seed coating without impairing germination in soybean and blackgram (Monisha et al., 2023). All three matrices meet the general requirements for seed-coating materials — biodegradability, low phytotoxicity, compatibility with microbial inoculants, and the capacity to maintain adhesion and moisture at the seed surface (Skrzypczak et al., 2021; Paravar et al., 2023; Wang et al., 2025). Chitosan was deliberately excluded despite its widespread use in seed treatment, as its intrinsic activity as a defense elicitor and antifungal agent would independently enhance resistance and disease protection, confounding attribution of any observed effect to the carrier matrix or to the encapsulated biocontrol agent (Jogaiah et al., 2020; Korbecka-Glinka et al., 2022).

The integration of functional biopolymers with endophytic fungi therefore represents a particularly attractive strategy for improving water and nutrient use efficiency, enhancing soil health, and increasing crop resilience while reducing reliance on synthetic agrochemicals (Yan et al., 2019; Gowtham et al., 2024). However, important knowledge gaps remain regarding the effects of polymeric seed-coating matrices on seed imbibition dynamics, their compatibility with endophytic propagules, and the extent to which encapsulation influences fungal survival, plant colonization, and biocontrol performance under realistic cultivation conditions (Tariq et al., 2023).

In tomato (*Solanum lycopersicum* L.), vascular wilt caused by *Fusarium oxysporum* f. sp. *lycopersici* (FOL) remains one of the most important phytosanitary constraints, with yield losses reaching up to 70% under severe disease pressure (Chaudhary et al., 2022; Muhorakeye et al., 2024). Several microbial agents, including endophytic fungi, have been evaluated for the biological control of FOL in tomato, among them *Trichoderma* spp., *Hypocrea lixii*, *Beauveria bassiana*, arbuscular mycorrhizal fungi, and certain yeasts (Pothiraj et al., 2021; Abdelaziz et al., 2022; Chaudhary et al., 2022; Devi et al., 2022; Nchu et al., 2022; Muhorakeye et al., 2024; Huertas et al., 2026). However, to current knowledge, the potential of dark septate endophytes for controlling FOL in tomato has not been specifically investigated. Although DSE have been recognized as promising plant growth-promoting and biocontrol-associated fungi in other crop–pathogen systems, their role in the tomato–FOL pathosystem remains largely unexplored, highlighting a relevant and timely research gap (De Lamo and Takken, 2020; Harsonowati et al., 2020; Santos et al., 2021; Mariani et al., 2024).

Accordingly, this study evaluated different biopolymers as components of tomato seed-coating systems, with emphasis on: (i) their effects on seed germination and seedling establishment; (ii) the viability and persistence of encapsulated endophytic fungal propagules; and (iii) the biocontrol of vascular wilt caused by FOL. By integrating material performance with biological functionality, this work aims to provide experimental evidence for the design of seed-coating formulations capable of improving disease management, input efficiency, and the sustainability of tomato production.

## 2 Materials and methods

### 2.1 Fungal strains, plant material, and biopolymers

Ten fungal strains with potential as biological control agents (BCAs) (**Table 1**) along with the phytopathogen FOL, were obtained from the culture collection of the Department of Agronomy at the University of Almería (Spain). For the *in vivo* assays, certified tomato seeds (Solanum lycopersicum L., cv. ‘ROMA VT-3831’) were provided by Mascarell Semillas (Valencia, Spain).

**Table 1:** Endophytic fungal isolates used in this study.

Code	Isolate	Source of endophyte/host plant	Reference
BC01	*Torula* sp.	–*	Huertas et al., 2026
BC06	*Sordaria fimicola*	*Hyparrhenia hirta*	Huertas et al., 2026
BC11	*Torula* sp.	–	Huertas et al., 2026
BC17	*Clarireedia calopus*	*Sedum sediforme*	Huertas et al., 2026
BC18	*Clarireedia calopus*	*Sedum sediforme*	Huertas et al., 2026
BC20	*Trichoderma gamsii*	*Genista umbellata*	Huertas et al., 2026
BC34	*Clarireedia narcissi*	*Genista umbellata*	Huertas et al., 2026
BC49	*Trichoderma saturnisporum*	*Anthyllis cytisoides*	Huertas et al., 2026
TA	*Trichoderma aggressivum* f.sp*. europaeum*	Mushroom with green mold	Sánchez-Montesinos et al., 2020
H1	*Trichoderma longibrachiatum*	*Posidonia oceanica*	Sánchez-Montesinos et al., 2019

* A dash (−) indicates that the host plant could not be identified at the time of collection.

For each isolate, the assigned code, the identified fungal species (or genus), the host plant or source of the endophyte, and the corresponding reference are indicated. (−) Indicates that the host plant could not be identified at the time of collection.

Three biopolymers were used as carrier matrices: sodium alginate (Cargill S.A., San Isidro, Buenos Aires, Argentina), with a molecular weight (Mw) of 1.97 × 10^5^ g mol^-^¹ and a mannuronate/guluronate (M/G) ratio of 0.6; gum arabic (Biopack, Argentina), with a Mw of 250,000 g mol^-^¹ and 99% purity; and guar gum (Cordis S.A., Argentina), with a Mw of 220,000 g mol^-^¹ and a mannose/galactose ratio of 1.8. To reduce the microbial load prior to use, the biopolymer powders were subjected to double exposure to UV light for 30 min per treatment. We note that UV irradiation acts on exposed surfaces and does not sterilise the interior of a bulk powder; autoclaving was deliberately avoided, since thermal treatment degrades alginate and alters the rheological properties of all three matrices, which would have confounded the comparison of film architecture central to this study. Following this surface decontamination step, the polymers were dissolved at the required concentrations and homogenized vigorously using a vortex mixer (Reax Top, Heidolph Instruments, Germany) prior to use to achieve complete hydration (Chin et al., 2021). The absence of viable culturable contaminants in the resulting solutions was verified by plating a 100 µL aliquot of each matrix onto nutrient agar (Merck, Germany) and incubating at 25 °C for 48 h; no microbial growth was recorded in any case prior to experimental use.

### 2.2 Biopolymer-seed compatibility test

The influence of biopolymer type, concentration, and imbibition time on seed germination and vigor was evaluated following the methodology described by Chin et al. (2021). Imbibition times of 15, 30 and 60 min were evaluated, covering the two shortest durations reported as optimal for biopolymer biopriming of vegetable seeds by Chin et al. (2021) plus a shorter level not previously examined. Biopolymer concentrations (**Table 2**) were based on ranges reported for seed coating and microbial encapsulation (Chin et al., 2021; Rocha et al., 2019) and adjusted to the concentration interval within which each polymer formed homogeneous, uniformly applicable solutions. Tomato seeds were first surface-disinfected with a 2% (w/v) sodium hypochlorite solution for 30 min, rinsed thoroughly, and dried under a laminar flow hood to ensure aseptic conditions. Seeds were then subjected to an imbibition process by immersion in the corresponding biopolymer solutions at the concentrations listed in (**Table 2**), after which they were dried on sterile filter paper within the laminar flow cabinet.

**Table 2:** Biopolymer concentrations (% w/v) and seed imbibition times (min) evaluated in the preliminary compatibility assays used to select the optimal carrier matrix for the biological control agents.

Biopolymers	Concentration % (w/v)	Seed imbibition time (min)
Sodium alginate (SA)	0.5; 1.0; 1.5; 2.0	
Gum arabic (GA)	25.0; 30.0; 35.0; 40.0	15; 30; 60
Guar gum (GG)	0.25; 0.50; 0.75; 1	

For the germination assays, treated seeds were placed in square Petri dishes (120 mm) containing a double layer of sterile filter paper, with moisture maintained through controlled irrigation with sterile distilled water. Three replicates were prepared per treatment, with ten seeds per plate. Germination percentage (GP) and ggermination rate index (GRI) were recorded every 48 h over a 5-day period under sterile laboratory conditions to prevent opportunistic contamination. Germination was strictly defined as the geotropic emergence of the radicle through the testa. The Germination Rate Index (GRI) was calculated according to the mathematical framework of Bhatt et al. (2015):


Germination rate index (GRI)=X1/Y1+(X2−X1)/Y2+⋯(Xn−(Xn−1))/Yn 

where X1, X2, …, Xn represent the number of seeds germinated on days 1, 2, …, n, respectively, and Y1, Y2, …, Yn represents the number of days from sowing to the first, second, …, nth count, respectively. GRI was used to determine the speed of germination under the different biopolymer treatments.

To assess early seedling fitness, the Seed Vigor Index (SVI) was calculated by integrating the allometric measurements of radicle and hypocotyl length, according to the following equation:


SVI=G%×(RL+HL)

where G% denotes the final germination percentage, RL represents the mean radicle length, and HL corresponds to the mean hypocotyl length. This index served as a comprehensive proxy for the physiological robustness of the seedlings following biopolymer treatment. The relative impact of the experimental variables was resolved through a multifactorial analysis of variance (ANOVA), designed to evaluate the main effects and second-order interactions among biopolymer identity, concentration, and imbibition time. Post-hoc comparisons were used to establish the definitive criteria for selecting the optimal imbibition parameters, ensuring maximum physiological performance for all subsequent antagonistic and biocontrol assays (Andrade et al., 2024).

### 2.3 Biopolymer-bioagent compatibility

A preliminary experiment was conducted to evaluate the influence of different biopolymer matrices on the viability of the selected biological control agents. The fungal isolates were cultured on potato dextrose agar (PDA; Merck, Germany) at 25 °C for seven days. Fungal propagules—including conidia, ascospores, and microsclerotia, depending on the isolate—were harvested by flooding the plate surfaces with sterile distilled water and gently dislodging the biomass with a sterile glass rod. The resulting suspensions were filtered, diluted, and incorporated into the respective biopolymer solutions (**Table 2**) to achieve a standardized final concentration of 10³ spores mL^-^¹. Three replicates were prepared per treatment. These mixtures were incubated under controlled conditions, and propagule viability was monitored at 24 and 48 h using a hemocytometer, determined as the number of germinated propagule mL^-^¹ at the end of the incubation period, following the methodology described by Chin et al. (2021).

The optimal concentration for each biopolymer matrix was selected based on the highest propagule viability recorded during these preliminary compatibility assays. Sodium alginate, gum arabic, and guar gum were evaluated at the specific concentrations detailed in **Table 2** to determine the most effective concentration for the subsequent encapsulation and inoculation stages. For all treatments, a control was established by applying 1 mL of sterile distilled water in lieu of the biopolymer-fungal formulations, providing a baseline for comparing the effects of the carrier matrices on fungal survival.

### 2.4 Biopolymer coating efficiency

Based on the preliminary viability assessments of conidia, ascospores, and microsclerotia (Section 2.3), integrated with the seed-biopolymer compatibility profiles (Section 2.2), an optimized formulation (matrix type, concentration, and imbibition kinetics) was established for each fungal taxon. The adherence efficiency and temporal stability of the coating were evaluated by quantifying the density of fungal propagules sequestered on the seed surface and their viability during cold storage (4 °C) at 24 h and at 10, 20, and 30 days post-coating.

To quantify the loading capacity of the biopolymer matrices, three replicate batches of five coated seeds each were prepared per fungal isolate and storage checkpoint. Each batch was immersed in 1 mL of sterile distilled water and subjected to vigorous vortexing to release the adhered biomass. Where propagule density exceeded the haemocytometer detection range, serial decimal dilutions were prepared in sterile distilled water and 100 µL of the appropriate dilution was spread-plated onto potato dextrose agar (PDA), incubated at 25 °C for 5 days, and colony-forming units were enumerated and back-calculated to CFU seed^-^¹. Propagule entrapment was determined from the number of surviving propagules recovered at the end of the incubation period (24 h) and calculated according to equation, adapted from Chin et al. (2021):


Propagule entrapment(%)=(Final viable propagule count)/(Initial viable propagule count)×100

The density of recovered propagules was determined microscopically using a haemocytometer. To assess the viability of the encapsulated fungi over the cold storage period, an aliquot of each suspension was serially diluted and plated on PDA at 24 h, 10, 20, and 30 days post-coating; colony-forming units (CFU seed^-^¹) were recorded after 5 days of incubation at 25 °C and expressed as log_10_ CFU seed^-^¹.

Scanning electron microscopy (SEM) was used to visualize the distribution of fungal spores, ascospores, and microsclerotia on bioprimed seeds. Five bioprimed seeds were randomly selected and dried directly in a desiccator containing silica gel for 48 h at room temperature. Before examination, seeds were sputter-coated with a 10-nm gold layer using a Leica EM ACE200 sputter coater. SEM analyses were performed using a high-resolution field emission scanning electron microscope (FE-SEM, Zeiss Sigma 300 VP) in high-vacuum mode.

### 2.5 Effect of biopriming on tomato seed germination and vigor after cold storage

Bioprimed tomato seeds were stored at 4 °C to evaluate whether biopriming with fungal inoculants affects germination performance and early seedling growth following cold storage. The experiment followed a completely randomized design with eleven treatments, comprising one biopriming treatment per fungal isolate—each applied using its individually optimized biopolymer formulation (Sections 2.2–2.4)—plus a non-bioprimed control lacking both the biopolymer carrier and the fungal inoculant. Three replicates were prepared per treatment, with five seeds per plate. At each storage checkpoint, germination percentage was recorded over a subsequent 5-day assay period under sterile conditions, and mean radicle length and mean hypocotyl length were measured after 10, 20, and 30 days of cold storage. The SVI was calculated at each storage checkpoint using the formula described in Section 2.2.

To assess tomato seed viability over the storage period, germination retention was evaluated from the initial timepoint until day 40 (Andrade et al., 2024). The area under the germination progress curve (AUC germination), indicative of cumulative germination capacity, was determined across the 10-, 20-, 30-, and 40-day storage intervals (Lahati et al., 2021). To evaluate the effect of biopriming treatments on seed physiological quality, a hierarchical cluster analysis (HCA) was performed based on four performance parameters: germination percentage, mean seedling vigor index (Mean SVI), germination retention (%), and germination area under the curve (Germination AUC). Raw data were normalized via Z-score transformation (global scaling) to enable cross-variable comparisons across different scales. The clustering algorithm was applied to the treatments (rows) using Euclidean distance as the dissimilarity metric and Ward’s method as the linkage criterion to identify functional clusters. Additionally, a global ranking index based on integrated Z-scores was calculated to determine the overall efficacy of each treatment relative to the control. The resulting high-resolution dendrogram-faced heatmap was generated to visualize phenotypic performance, where positive (green) and negative (red) Z-scores denoted superior and inferior seed performance, respectively.

### 2.6 Effectiveness of biocontrol agents applied via seed biopriming versus substrate inoculation in pots

The efficacy of the biocontrol agents (BCA) against the pathogen (*Fusarium oxysporum* f. sp. *lycopersici*, FOL) was evaluated by comparing two application methods. For the substrate inoculation treatment, three 5-mm mycelial discs of each BCA were placed into 100-mL pots containing vermiculite previously moistened to container capacity. These pots were sealed with aluminium foil and incubated at 25 °C for 7 days to ensure thorough substrate colonization by the fungal isolates. Concurrently, tomato seeds were pre-germinated in a humid chamber at 25 °C in darkness and subsequently transplanted into the colonized pots (one seed per pot). For the seed biopriming treatment, BCA-primed seeds were pre-germinated and transplanted into 100-mL pots of sterile vermiculite under identical environmental conditions. A control group was maintained, consisting of seedlings that received no BCA treatment (neither via biopriming nor substrate inoculation).

The experiment followed a completely randomized design with three replicates per treatment, each comprising 10 pots (one seedling per pot), totaling 30 plants per treatment. Fourteen days post-transplantation, or upon the development of two true leaves, all seedlings were challenged with FOL. During the vegetative stage, the chlorophyll index (SPAD) was monitored periodically throughout the experiment, and results corresponding to 23 DAI — the timepoint at which maximum differentiation among treatments was observed — are presented. At the end of the experiment, plant height, fresh and dry weight, seedling diameter, number of leaves, and leaf area were measured.

Disease symptoms were monitored periodically, and the results presented correspond to 23 DAI, when disease severity differences among treatments were most pronounced. Disease severity was scored using a 1-to-5 ordinal scale: 1, no visible disease symptoms; 2, slight infection (<20% of leaves with chlorosis, 25% wilting); 3, moderate infection (20–50% of leaves with chlorosis, 50% wilting); 4, extensive infection (50–80% of leaves with chlorosis, 75% wilting); 5, severe infection (80–100% of leaves with chlorosis, 100% wilting, leading to plant death). Disease incidence and disease severity (Biswas et al., 2012) were calculated using the following formulas:


Disease incidence(DI,%)=(number of infected plants/total number of plants assessed)×100

Disease severity(DS,%), or Infection Index=[sum of all disease ratings/(total number of ratings×maximum disease score)]×100

### 2.7 Statistical analysis

All statistical analyses were performed using Statgraphics Centurion, version 19.1.3 (Statgraphics Technologies, Inc., The Plains, VA, USA), except for the area under the germination progress curve (AUC) and viable fungal propagules on bioprimed seeds, which were calculated using custom scripts in R (v. 4.2.0; R Core Team, 2022). Data normality and homogeneity of variance were verified prior to analysis using the Shapiro–Wilk and Levene’s tests, respectively. For the biopolymer-seed compatibility assays (Section 2.2), experimental data were analyzed using multifactorial analysis of variance (ANOVA) to assess the main effects of biopolymer type, concentration, and imbibition time, as well as their second-order interactions. For the biopolymer-bioagent compatibility assays (Section 2.3) and the biopolymer coating efficiency assays (Section 2.4), one-way or multifactorial ANOVA was applied, as appropriate, to evaluate the effects of biopolymer matrix, fungal isolate identity, and storage time on propagule viability, entrapment efficiency, and RSR. For the effect of biopriming on seed germination and vigor after cold storage (Section 2.5), a hierarchical cluster analysis (HCA) based on Z-score-normalized performance parameters (germination percentage, Mean SVI, germination retention, and Germination AUC) was performed using Euclidean distance as the dissimilarity metric and Ward’s method as the linkage criterion, using R. For the biocontrol efficacy assays (Section 2.6), the main effects of application method (seed biopriming vs. substrate inoculation) and fungal isolate identity on plant growth parameters, SPAD index, disease incidence, and disease severity were evaluated using two-way ANOVA; disease incidence and severity data, expressed as percentages, were arcsine-square-root transformed prior to analysis to meet normality assumptions. When significant differences were detected in any of the above analyses, Tukey’s Honestly Significant Difference (HSD) *post-hoc* test was applied for pairwise comparisons (p ≤ 0.05) (Chakraborty et al., 2023; Singh et al., 2020). All data are expressed as mean ± standard deviation (SD).

## 3 Results

### 3.1 Biopolymer-seed compatibility

Imbibition time was the determining factor in the germination response of tomato seeds, overriding the influence of biopolymer type or concentration (**Table 3**). Imbibition at 30 min proved incompatible with germination across all treatments evaluated, reducing germination percentage (GP), germination rate index (GRI), and seed vigor index (SVI) to near-zero values in all cases, with statistically significant differences compared to the 15- and 60-min imbibition times (p< 0.05).

**Table 3:** Effect of imbibition time (15, 30, and 60 min) on Germination Percentage (GP), Germination Rate Index (GRI), and Seed Vigor Index (SVI) of tomato seeds treated with different biopolymer matrices and concentrations.

Treatment	Parameter	15 min	30 min	60 min	F	p-value
Control	GP	90.33 ± 17.71a	2.33 ± 7.28b	87.00 ± 24.09a	236.38	<0.001
	GRI	4.99 ± 2.05a	0.04 ± 0.11b	5.58 ± 2.01a	101.03	<0.001
	SVI	526.93 ± 216.24a	7.53 ± 23.67b	508.37 ± 185.68a	95.53	<0.001
SA (0.5%)	GP	100.00 ± 0.00a	4.00 ± 9.32c	90.00 ± 14.38b	853.25	<0.001
	GRI	7.87 ± 1.17a	0.06 ± 0.14c	5.67 ± 1.68b	345.55	<0.001
	SVI	577.00 ± 205.71a	14.23 ± 32.94b	499.03 ± 155.59a	123.76	<0.001
SA (1%)	GP	90.00 ± 0.00b	0.00 ± 0.00c	100.00 ± 0.00a	>1000	<0.001
	GRI	6.56 ± 1.69a	0.00 ± 0.00c	5.78 ± 0.64b	353.10	<0.001
	SVI	584.70 ± 148.46a	0.00 ± 0.00b	530.67 ± 185.29a	166.68	<0.001
SA (1.5%)	GP	90.67 ± 8.28a	0.33 ± 1.83b	86.67 ± 20.14a	537.27	<0.001
	GRI	6.58 ± 0.41a	0.00 ± 0.03c	5.61 ± 1.32b	668.97	<0.001
	SVI	567.93 ± 138.17a	0.40 ± 2.19b	511.21 ± 215.78a	142.47	<0.001
SA (2%)	GP	86.67 ± 4.79a	0.33 ± 1.83b	87.00 ± 16.43a	757.52	<0.001
	GRI	6.44 ± 1.31a	0.01 ± 0.04b	6.37 ± 1.46a	319.45	<0.001
	SVI	541.53 ± 108.86a	1.17 ± 6.39b	568.50 ± 186.60a	197.36	<0.001
GA (25%)	GP	83.33 ± 9.59a	0.00 ± 0.00b	90.00 ± 24.91a	317.60	<0.001
	GRI	5.67 ± 0.55b	0.00 ± 0.00c	6.56 ± 2.12a	236.76	<0.001
	SVI	516.10 ± 137.75a	0.00 ± 0.00b	511.07 ± 213.40a	122.66	<0.001
GA (30%)	GP	87.00 ± 4.66a	7.00 ± 12.08b	93.33 ± 18.26a	416.04	<0.001
	GRI	4.77 ± 0.92b	0.12 ± 0.21c	6.53 ± 1.62a	281.96	<0.001
	SVI	447.57 ± 176.19b	28.40 ± 68.63c	579.80 ± 215.33a	90.82	<0.001
GA (35%)	GP	96.67 ± 4.79a	7.33 ± 13.37c	75.00 ± 30.26b	174.90	<0.001
	GRI	7.67 ± 0.48a	0.10 ± 0.19c	6.54 ± 2.62b	210.92	<0.001
	SVI	652.10 ± 109.02a	25.60 ± 54.01c	465.53 ± 222.98b	144.33	<0.001
GA (40%)	GP	93.33 ± 9.59a	10.67 ± 17.01b	90.00 ± 14.38a	335.14	<0.001
	GRI	7.11 ± 0.85a	0.18 ± 0.28c	5.44 ± 0.89b	742.55	<0.001
	SVI	599.33 ± 180.95a	27.13 ± 49.17b	506.90 ± 244.05a	89.65	<0.001
GG (0.25%)	GP	93.33 ± 9.59a	4.33 ± 7.74c	49.00 ± 34.78b	130.94	<0.001
	GRI	5.33 ± 0.55a	0.07 ± 0.14c	3.19 ± 2.52b	94.32	<0.001
	SVI	500.00 ± 215.57a	12.20 ± 23.89c	288.03 ± 237.69b	52.01	<0.001
GG (0.5%)	GP	76.67 ± 4.79a	2.67 ± 6.40b	87.00 ± 29.84a	199.48	<0.001
	GRI	5.40 ± 0.94a	0.05 ± 0.11b	5.70 ± 2.05a	177.89	<0.001
	SVI	442.93 ± 89.07a	8.43 ± 22.86b	517.93 ± 216.83a	122.77	<0.001
GG (0.75%)	GP	86.67 ± 4.79b	4.33 ± 9.35c	96.67 ± 4.79a	>1000	<0.001
	GRI	7.11 ± 0.80a	0.08 ± 0.16b	6.78 ± 1.76a	377.75	<0.001
	SVI	608.23 ± 99.22a	18.07 ± 42.04b	547.67 ± 215.09a	163.92	<0.001
GG (1%)	GP	90.00 ± 8.30a	7.00 ± 13.68b	77.67 ± 33.80a	129.05	<0.001
	GRI	5.67 ± 0.28a	0.13 ± 0.25b	5.12 ± 2.11a	182.39	<0.001
	SVI	349.20 ± 244.74a	25.57 ± 54.94b	366.17 ± 199.97a	32.22	<0.001

Values are expressed as mean ± standard deviation (n = 3). Different lowercase letters within a row indicate statistically significant differences among imbibition times (15, 30, and 60 min) within the same treatment, according to Tukey’s honest significant difference (HSD) *post-hoc* test (p< 0.05). F- and p-values correspond to one-way ANOVA performed independently for each treatment. GP, Germination Percentage (%); GRI, Germination Rate Index; SVI, Seed Vigor Index; SA, sodium alginate; GA, gum arabic; GG, guar gum.

In contrast, imbibition times of 15 and 60 min maintained seed viability and vigor at satisfactory levels across most treatments. At 15 min, the highest and most consistent results were obtained, with GP values exceeding 80% for all biopolymers and concentrations evaluated. At 60 min, results were comparable for most treatments, although GG (0.25%) and GA (35%) showed significant reductions in GP and SVI, indicating reduced compatibility at longer exposure times with these particular formulations.

Based on these results, 15 min was selected as the optimal imbibition time for all subsequent assays. Regarding the biopolymer matrix, SA (0.5–1%) showed the highest and most consistent germinative performance, with GP, GRI, and SVI values equivalent to or higher than the control across all tested conditions. GA (25–40%) also yielded satisfactory results at 15 min, with GP values exceeding 80% at all concentrations evaluated and the highest GRI and SVI values recorded at GA (35%). GG showed a more heterogeneous behavior, with notable reductions at 60 min at the lowest concentrations tested. Therefore, based solely on seed compatibility criteria, SA and GA at 15 min imbibition were identified as the matrices with the greatest potential for subsequent assays.

### 3.2 Biopolymer-bioagent compatibility: effects on fungal propagule viability

**Figure 1:**
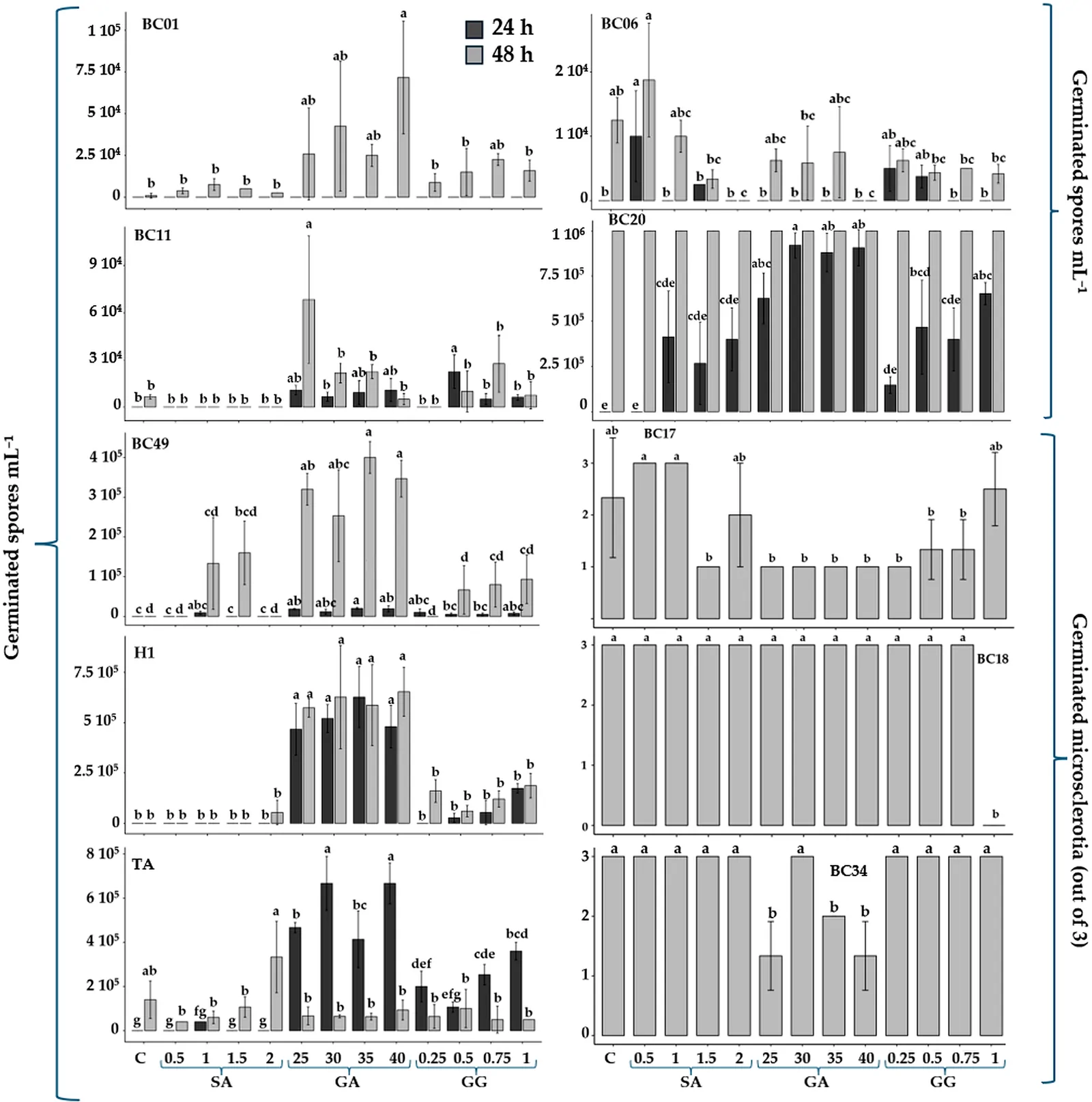
Effect of biopolymer matrix and concentration on the germination/viability of fungal propagules from ten biological control agent isolates (BC01, BC06, BC11, BC20, BC49, H1, TA, BC17, BC18 and BC34) at 24 h (dark grey bars) and 48 h (light grey bars) of incubation. For isolates BC01, BC06, BC11, BC20, BC49, H1, and TA, viability was assessed as the number of germinated spores mL^-^¹ (left axis). For isolates BC17, BC18 and BC34, viability was assessed as the number of germinated microsclerotia, out of a total of 3 microsclerotia evaluated per treatment (right axis). C, control (sterile distilled water); SA, sodium alginate (0.5, 1, 1.5, and 2% w/v); GA, gum arabic (25, 30, 35, and 40% w/v); GG, guar gum (0.25, 0.5, 0.75, and 1% w/v). Bars represent mean values ± standard deviation. Different letters above bars indicate statistically significant differences between treatments according to Tukey’s test (p< 0.05).

The viability of fungal propagules varied considerably depending on the biopolymer matrix, concentration, and isolate evaluated (**Figure 1**). For most spore-producing isolates (BC01, BC11, BC20, BC49, H1, and TA), gum arabic (GA) yielded the highest germination rates, generally at intermediate-to-high concentrations (25–40% w/v), and outperformed both sodium alginate (SA) and guar gum (GG) across the assay. Notably, BC20 and H1 reached the highest absolute germination values (~9 × 10^5^ and ~6.5 × 10^5^ spores mL^-^¹, respectively) when formulated with GA, whereas SA resulted in negligible or undetectable germination for several isolates (e.g., BC11, BC20, and H1). In contrast, BC06 was the only spore-producing isolate for which SA (0.5% w/v) outperformed GA, achieving the highest germination at 48 h.

For isolates producing microsclerotia (BC17, BC18, and BC34), the pattern differed markedly from that observed for spore-forming isolates. Overall viability remained high (≥1 out of 3 microsclerotia germinated) across most treatments, with BC18 showing the broadest compatibility, retaining maximum germination (3/3) under nearly all biopolymer-concentration combinations except GG at 1% w/v. For BC17, SA (0.5–1% w/v) and GG (1% w/v) preserved germination comparable to the control, while for BC34, GA significantly reduced viability at all tested concentrations (25–40% w/v), in clear contrast to its positive effect on spore-producing isolates.

Taken together, these results indicate that propagule type strongly influenced biopolymer compatibility: spore-producing isolates generally benefited from GA at medium-to-high concentrations, whereas microsclerotia-producing isolates exhibited greater tolerance to SA and GG, with GA proving detrimental in at least one case (BC34). Based on these findings, GA (25–40% w/v, isolate-dependent) was selected as the optimal carrier for spore-producing isolates, while SA (0.5–1% w/v) was selected for microsclerotia-producing isolates for use in subsequent encapsulation and inoculation assays. The selected biopolymer matrix, optimal concentration, and incubation time for each isolate are summarized in **Table 4**.

**Table 4:** Optimized biopolymer formulation parameters selected for each fungal biocontrol isolate based on maximum propagule viability.

Code	Propagule type	Selected biopolymer	Optimal concentration (% W/V)	Optimal incubation time (h)
BC01	Spores	GA	40	48
BC06	Spores	SA	0.5	48
BC11	Spores	GA	25	48
BC17	Microsclerotia	SA	0.5	–
BC18	Microsclerotia	SA	0.5	–
BC20	Spores	GA	30	48
BC34	Microsclerotia	SA	0.5	–
BC49	Spores	GA	35	48
TA	Spores	GA	30	24
H1	Spores	GA	30	24–48

Gum arabic (GA); Sodium Alginate (SA). Selections were based on the highest fungal propagule viability (germinated spores mL^-^¹ or germinated microsclerotia (out of 3) recorded in the preliminary biopolymer-bioagent compatibility assays (**Figure 1**), determined by Tukey’s test (p< 0.05).

### 3.3 Propagule entrapment and viability of bioprimed seeds during cold storage

**Figure 2:**
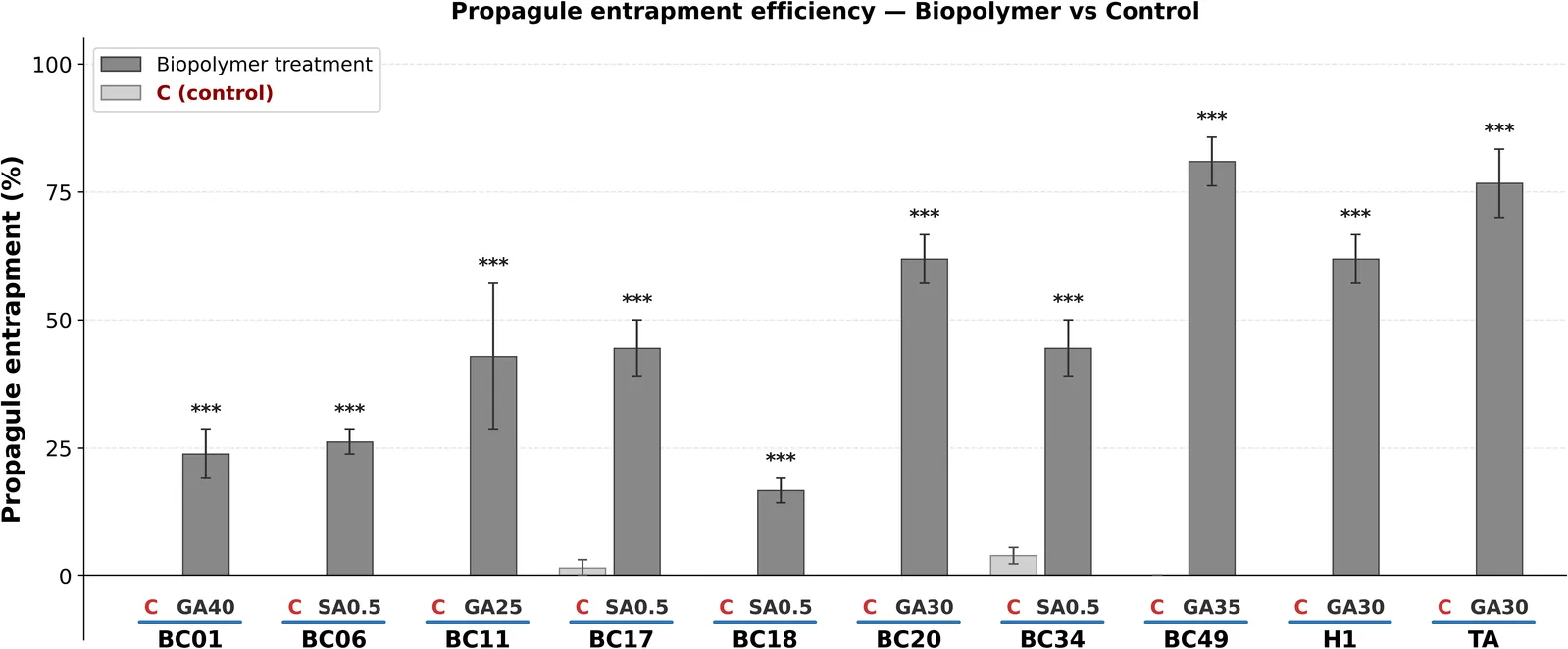
Propagule entrapment (%) of fungal biocontrol isolates coated with their optimal biopolymer formulation at 24 h post-coating. C, control (sterile distilled water); GA, gum arabic (25, 35, or 40% w/v); SA, sodium alginate (0.5% w/v). Bars represent mean values ± standard deviation (n = 3). Asterisks indicate statistically significant differences between the biopolymer treatment and its corresponding control according to Tukey’s test (***p ≤ 0.001).

The incorporation of biopolymer matrices markedly improved fungal propagule retention at the seed surface, while control treatments showed negligible or undetectable adhesion in virtually all isolates evaluated (**Figure 2**). Propagule entrapment varied considerably depending on the isolate and the formulation applied, suggesting that propagule retention is driven by specific interactions between the polymer matrix and the fungal cell surface. Gum arabic was the carrier that achieved the highest entrapment values for spore-producing isolates. BC49 with GA at 35% recorded the highest entrapment in the study (81.2%), while TA and H1, both with GA at 30%, yielded values of 76.8% and 62.1%, respectively, and BC20 with GA at 30% reached 62.0%. However, higher gum arabic concentration did not guarantee greater retention: BC01 with GA at 40% reached only 23.9%, well below BC20 and BC49, indicating that the relationship between polymer concentration and propagule retention is non-linear and isolate-dependent. For isolates formulated with sodium alginate at 0.5%, results were moderate but statistically significant. BC17 and BC34 reached similar values (44.0% and 44.4%, respectively), substantially above their controls, of which only BC34 showed detectable residual adhesion (3.9%). BC06 achieved 26.0% and BC18 recorded the lowest value in the study (16.7%). In all remaining control treatments no adhesion was detected, confirming that biopolymer presence is essential for fungal retention under the experimental conditions evaluated.

**Figure 3:**
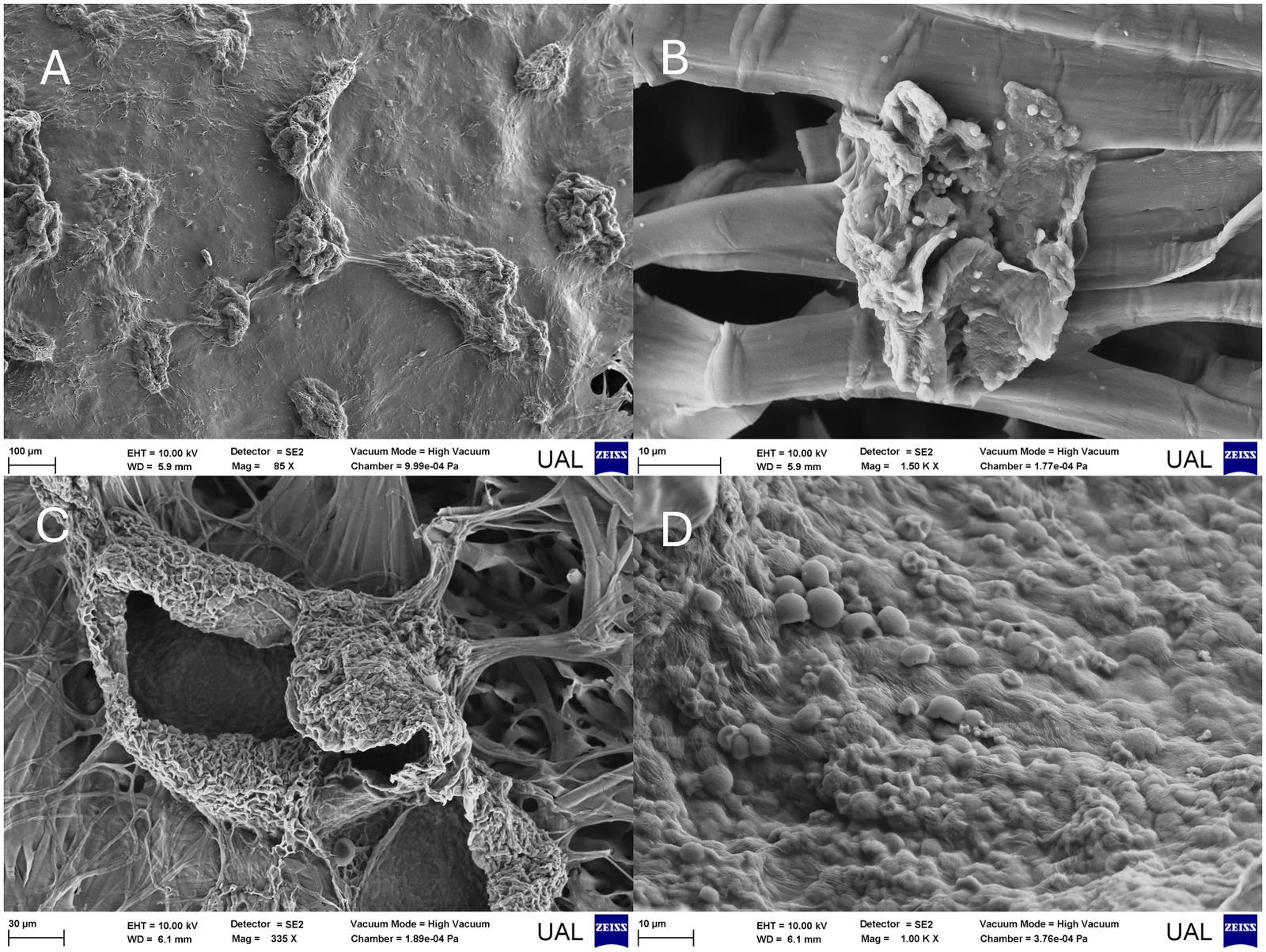
Scanning electron microscopy (FE-SEM) images of bioprimed tomato seeds showing fungal propagule distribution and biopolymer coating morphology. **(A)** Clarireedia calopus coated with sodium alginate (SA, 0.5% w/v); microsclerotia interconnected by hyphal cords anchored to the alginate film (scale bar = 100 µm; magnification 85×). **(B)** Torula sp. coated with sodium alginate (SA, 0.5% w/v); spherical to semi-spherical conidia (1.0–2.0 µm) embedded within the polymer network (scale bar = 10 µm; magnification 1.50 K×). **(C)** Sordaria fimicola coated with sodium alginate (SA, 0.5% w/v); globose perithecium partially collapsed under high-vacuum conditions, anchored by the alginate network (scale bar = 30 µm; magnification 335×). **(D)** Trichoderma saturnisporum coated with gum arabic (GA, 35% w/v); globose to subglobose conidia (2.0–4.0 µm) firmly anchored within the gum arabic matrix (scale bar = 10 µm; magnification 1.00 K×). All images were acquired at 10.00 kV in high-vacuum mode using a Zeiss Sigma 300 VP field emission scanning electron microscope.

Scanning electron microscopy (SEM) analysis of the tomato seed surface allowed visualization of the biopolymer coating distribution and the establishment of the different biocontrol agents following biopriming. In seeds treated with sodium alginate at 0.5% and *Clarireedia calopus* (BC17) (**Figure 3A**), the biopolymer formed a continuous, homogeneous film covering the natural irregularities of the seed tegument, acting as a three-dimensional physical support that favored fungal anchoring. Over this protective layer, multiple microsclerotia in advanced stages of organization were observed, displaying their characteristic irregular, globose morphology with a markedly corrugated surface resulting from dense cellular packing. Notably, adjacent microsclerotia were not isolated but actively interconnected by thick hyphal cords extending across the alginate film, demonstrating that this polymer concentration not only proved biocompatible but also provided a microenvironment conducive to active hyphal growth and intercellular communication at the seed surface.

In seeds treated with sodium alginate at 0.5% and *Torula* sp. (BC01 and BC11) (**Figure 3B**), fungal propagules were identified as numerous spherical to semi-spherical cells of approximately 1.0–2.0 µm in diameter, partially embedded within the polymer network and concentrated predominantly in the microtopographic depressions of the testa. The alginate formed a reticular, porous, multilayer film that adapted to the seed surface microtopography and supported a high-density colonization, evidenced by an extensive network of interwoven fungal hyphae anchored through the polymer scaffold. Additionally, a *Sordaria fimicola* (BC06) perithecium was clearly distinguished — a prominent, globose ascoma partially collapsed under high-vacuum conditions, densely surrounded by mycelial cords and firmly anchored by the alginate network, confirming the active establishment and development of the biocontrol agent directly on the seed surface (**Figure 3C**).

In seeds treated with gum arabic at 35% and *Trichoderma* sp. (**Figure 3D**), the biopolymer formed a dense, uniform, and continuous coating that masked the defined cellular boundaries of the testa, creating a cohesive organic matrix. High-resolution analysis confirmed efficient immobilization of the biocontrol agent: conidia were identified as grouped and individual propagules with characteristic globose to subglobose morphologies ranging from approximately 2.0 to 4.0 µm in diameter, firmly anchored to the surface and partially embedded within the microfolding of the gum arabic matrix, with no signs of structural collapse.

**Figure 4:**
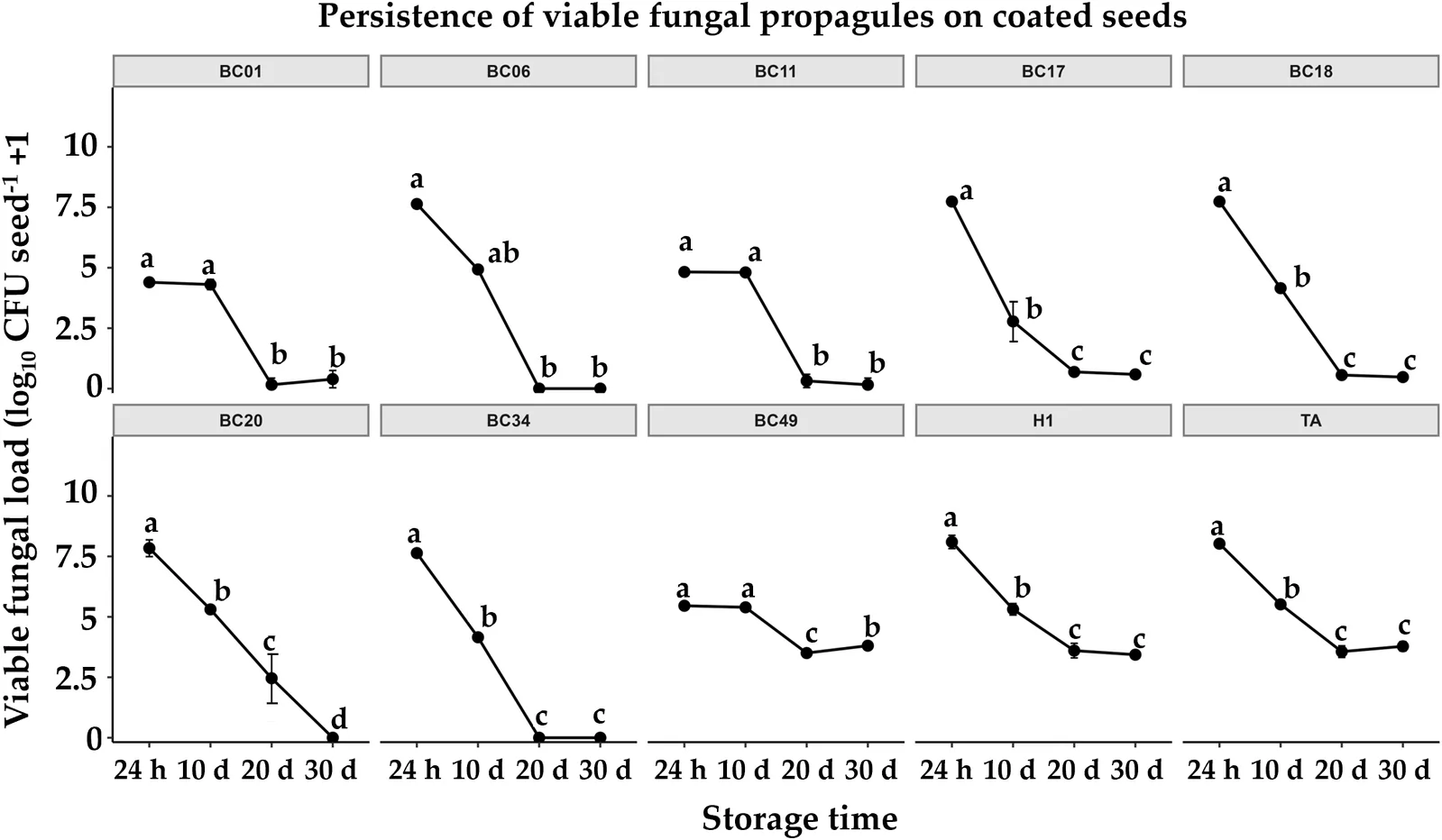
Persistence of viable fungal propagules on bioprimed tomato seeds stored at 4 °C over a 30-day period. The viable fungal load is expressed as log_10_ (CFU seed^-^¹ + 1). Each isolate was formulated with its individually optimized biopolymer carrier (**Table 4**). Measurements were taken at 24 h, 10, 20, and 30 days post-coating. Points represent mean values ± standard deviation (n = 3). Different lowercase letters indicate statistically significant differences among storage time-points within each isolate according to Tukey’s HSD test (p ≤ 0.05).

**Figure 4** shows the persistence of viable fungal propagules on coated seeds over a 30-day storage period. All isolates showed a general declining trend, although with notable differences in the magnitude and rate of viability loss. Isolates BC06, BC20 and BC34 showed the most pronounced declines, reaching undetectable or near-zero loads by the end of the period. BC20 stood out for showing statistically significant differences among all sampling time-points, being the only isolate with a continuous and progressive reduction throughout the entire storage period. BC17 and BC18 showed equally pronounced declines, stabilising at low residual values from day 20 onwards. H1 and TA followed a very similar pattern to each other, with significant decline until day 20 and subsequent stabilisation, maintaining detectable residual loads at the end of the period. BC01 and BC11 started from moderate loads and showed significant declines from days 10 and 20 respectively. BC49 was the most stable isolate, with no significant differences between 24 h and day 10, showing a later and more moderate decline than the rest.

**Figure 5:**
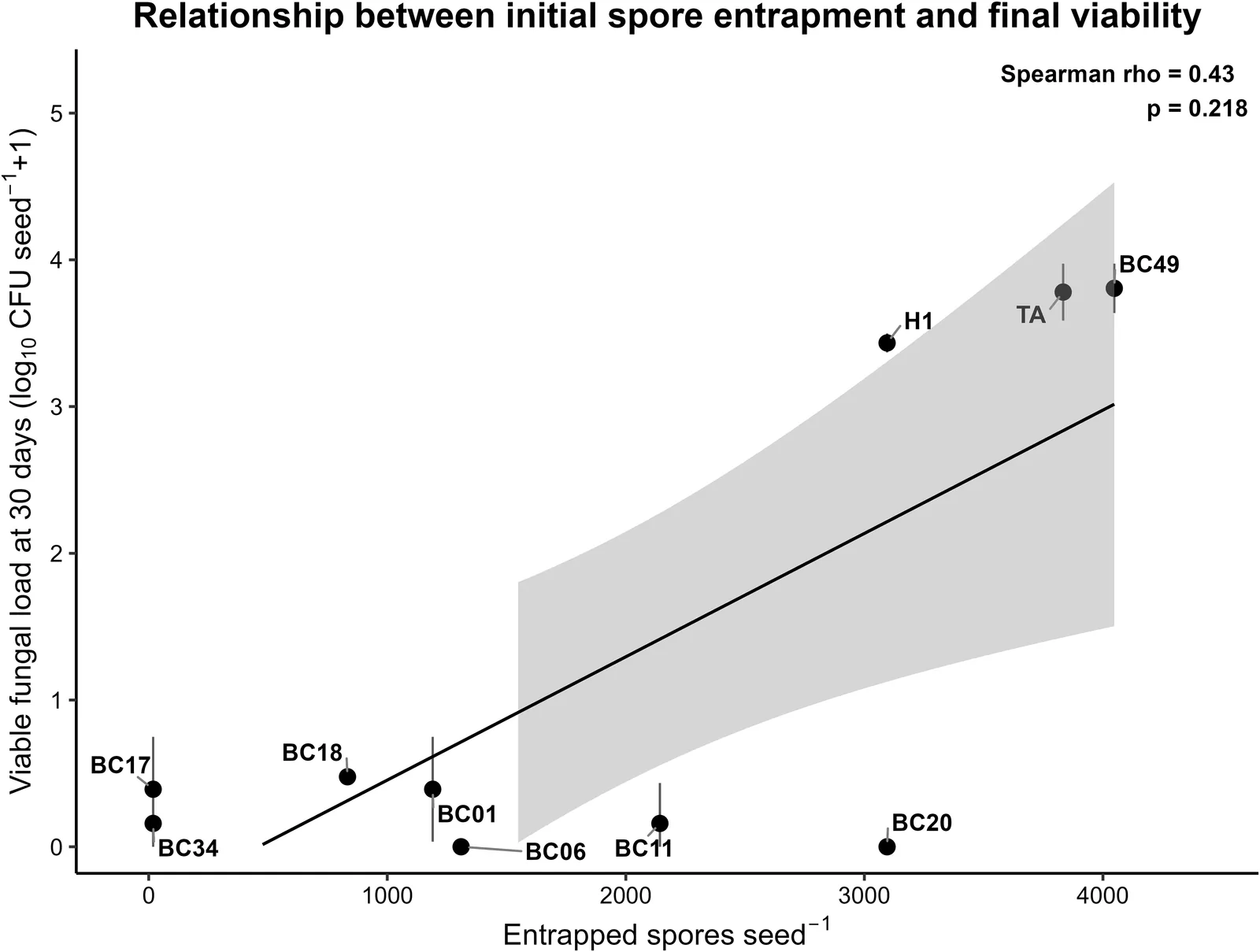
Relationship between initial propagule entrapment efficiency and viable fungal load after 30 days of cold storage (4 °C) for the ten bioprimed isolates. The x-axis represents the number of entrapped spores per seed (spores seed^-^¹) and the y-axis represents the viable fungal load recovered at 30 days expressed as log_10_ CFU seed^-^¹ +1. Error bars indicate standard error of the mean (n = 3). The solid line represents the linear regression fit and the shaded area indicates the 95% confidence interval. Spearman rank correlation coefficient (rho) and associated p-value are shown in the upper right corner.

The relationship between initial propagule entrapment efficiency and long-term viability after 30 days of cold storage was assessed through Spearman correlation analysis (**Figure 5**) and was not statistically significant (rho = 0.43, p = 0.218). Isolates BC49, TA, and H1, which presented the highest entrapment values (>3,000 spores seed^-^¹), also retained the highest viable fungal loads at 30 days (~3.4–3.8 log_10_ CFU seed^-^¹ +1). In contrast, isolates BC17 and BC34 showed negligible entrapment (<100 spores seed^-^¹) yet retained detectable viable propagules at 30 days, indicating that viability persistence is not exclusively determined by initial loading capacity and may be influenced by intrinsic differences in propagule resilience among fungal taxa. Similarly, isolates BC06, BC11, and BC20, despite moderate to high entrapment values, showed near-zero viability at 30 days, suggesting that the nature of the propagule type and its compatibility with the biopolymer matrix may play a more decisive role in long-term survival than entrapment efficiency alone. Taken together, these results highlight that initial spore entrapment is a necessary but not sufficient condition for ensuring propagule persistence during cold storage, and that isolate-specific biological traits must be considered when designing effective biopriming formulations.

### 3.4 Germination and vigor of bioprimed tomato seeds after cold storage

**Figure 6:**
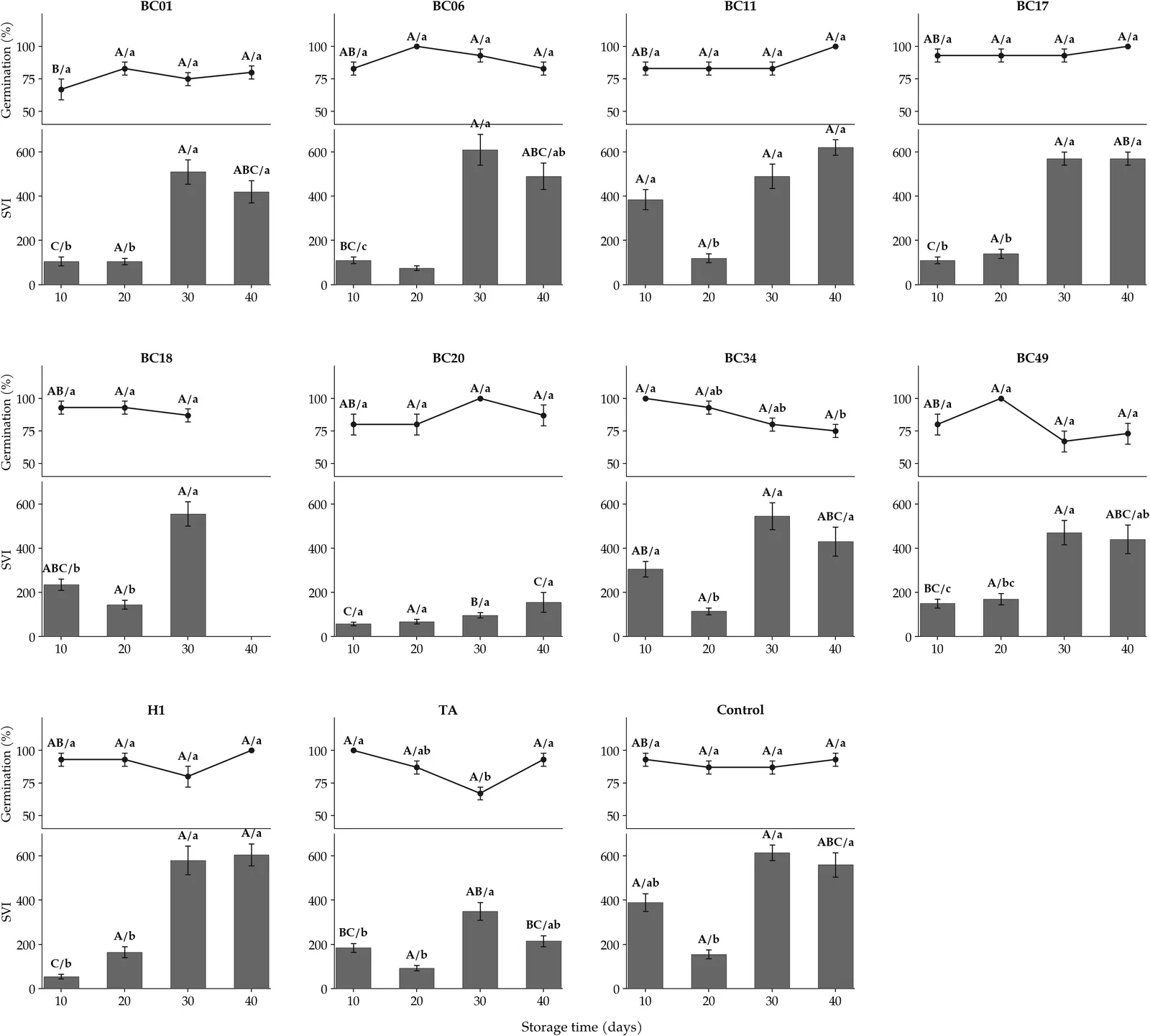
Germination percentage (upper panels) and seedling vigor index (SVI, lower panels) of bioprimed tomato seeds stored at 4 °C for up to 40 days. Each panel represents an individual fungal isolate or the non-bioprimed control. Values are means ± standard error (n = 3). Uppercase letters indicate significant differences among storage timepoints within each treatment; lowercase letters indicate significant differences among treatments at each timepoint (Tukey’s HSD test, p< 0.05).

Germination percentage and seedling vigor index (SVI) of bioprimed tomato seeds were monitored at 10, 20, 30, and 40 days of cold storage (4 °C) to assess the effect of fungal biopriming on seed performance over time. Results for all isolates and the non-bioprimed control are shown in **Figure 6**.

Germination remained stable and high across all treatments throughout the storage period, with values generally above 75%. Only BC01 showed lower germination at the beginning of storage (day 10), recovering at subsequent timepoints. BC34 was the only isolate with a sustained decreasing trend over time. TA experienced a transient decline at day 30, recovering by day 40. In no case did biopriming significantly reduce germination capacity compared to the non-bioprimed control. The SVI showed a more variable dynamic among isolates. Most treatments exhibited low values during the first weeks of storage, with a marked increase from day 30 onwards, particularly in BC01, BC06, BC17, BC49, and H1. In contrast, BC11, BC34, and the control maintained relatively stable vigor from the beginning of storage. BC18 followed a similar recovery pattern up to day 30, with no data available at day 40. TA showed the most irregular pattern, with a decline at day 20 followed by partial recovery. BC20 consistently presented the lowest SVI values of all treatments throughout the entire storage period, although without significant differences compared to the control.

**Figure 7:**
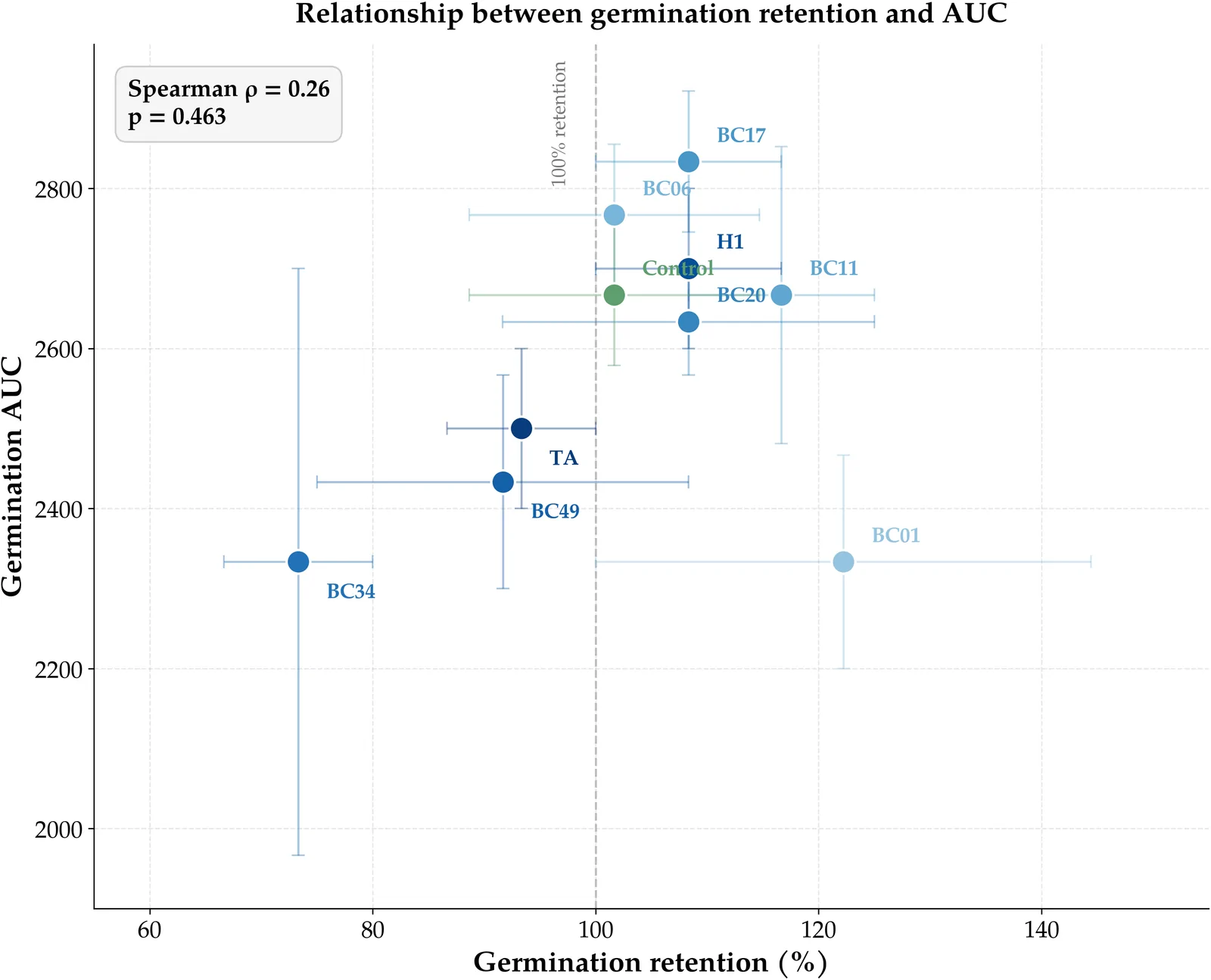
Germination retention (%, upper panel) and area under the germination progress curve (AUC germination, lower panel) of bioprimed tomato seeds stored at 4 °C for up to 40 days. The dashed line indicates 100% germination retention. Values are means ± standard error (n = 3). No statistically significant differences were detected among treatments for either variable.

To further assess the effect of cold storage on seed viability, germination retention and the area under the germination progress curve (AUC) were calculated for each biopriming treatment, and their relationship is shown in **Figure 7**. Germination retention values were close to or above 100% in most treatments, indicating that cold storage did not negatively affect seed viability over the 40-day period. BC01 and BC11 showed the highest retention values (~122% and ~117% respectively), suggesting a slight stimulatory effect of storage on germination capacity, whereas BC34 showed the lowest retention (~73%), indicating a progressive loss of germinability over time. Regarding germination AUC, values were similar across all treatments, ranging approximately from 2330 to 2830, with BC17 and BC06 showing the highest cumulative germination capacity and BC01 and BC34 the lowest. No statistically significant differences were detected among treatments for either parameter (ANOVA, p = 0.376 for retention and p = 0.329 for AUC). A Spearman correlation analysis between germination retention and AUC was not statistically significant (rho = 0.26, p = 0.463), indicating that the two parameters captured largely independent aspects of seed germination performance under cold storage rather than being redundant measures of the same trait.

**Figure 8:**
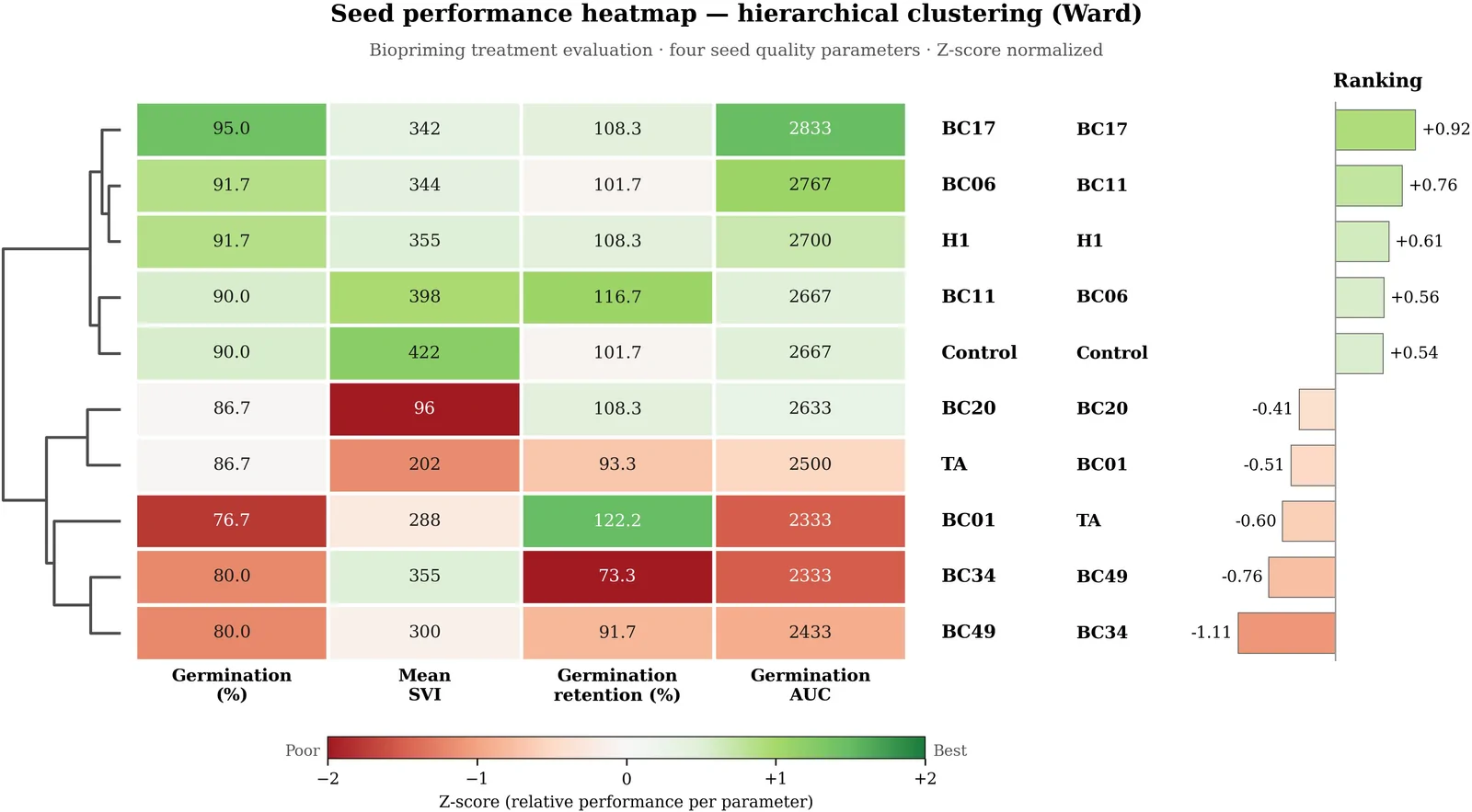
Heatmap of seed quality parameters across biopriming treatments after cold storage (4 °C, 40 days). Rows represent fungal isolates ordered by Ward hierarchical clustering (dendrogram, left). Columns represent four seed quality parameters: germination percentage (mean across storage timepoints), mean seedling vigor index (SVI), germination retention (%), and area under the germination progress curve (AUC). Cell values show original means; cell color indicates the z-score normalized value relative to the global mean of each parameter (red: below average; white: average; green: above average). The ranking panel (right) shows the overall performance score per isolate, calculated as the mean z-score across all four parameters. BC18 was excluded owing to the absence of data at day 40.

Hierarchical clustering analysis revealed two main clusters among the evaluated biopriming treatments (**Figure 8**). The first cluster grouped BC17, BC06, H1, BC11, and the non-bioprimed control, characterized by high mean SVI and germination AUC values. The second cluster included BC01, BC49, BC20, BC34, and TA, showing a more heterogeneous profile with generally lower vigor values. Regarding individual variables, germination percentage showed little variability among treatments, with all isolates close to the global mean. The most discriminating variable was mean SVI, where BC20 showed the lowest value of all treatments (~97), in contrast to H1, BC11, BC06, and the control, which showed the highest values (~355–505). Concerning germination retention, BC01 and BC11 exceeded 100% (~122% and ~117% respectively), suggesting a slight stimulatory effect of cold storage on germination capacity, while BC34 was the only treatment showing a clear loss of viability (~73%). Germination AUC showed less variability among treatments, with BC17 and BC06 presenting the highest values (~2833 and ~2767) and BC01 and BC34 the lowest (~2333). The overall ranking based on the mean z-score across the four variables identified BC17, BC06, and H1 as the best-performing treatments for seed conservation under cold storage, whereas BC20, BC34, and BC01 showed the least favorable overall profile.

### 3.5 Biocontrol efficacy and plant performance under substrate inoculation and seed biopriming

**Figure 9:**
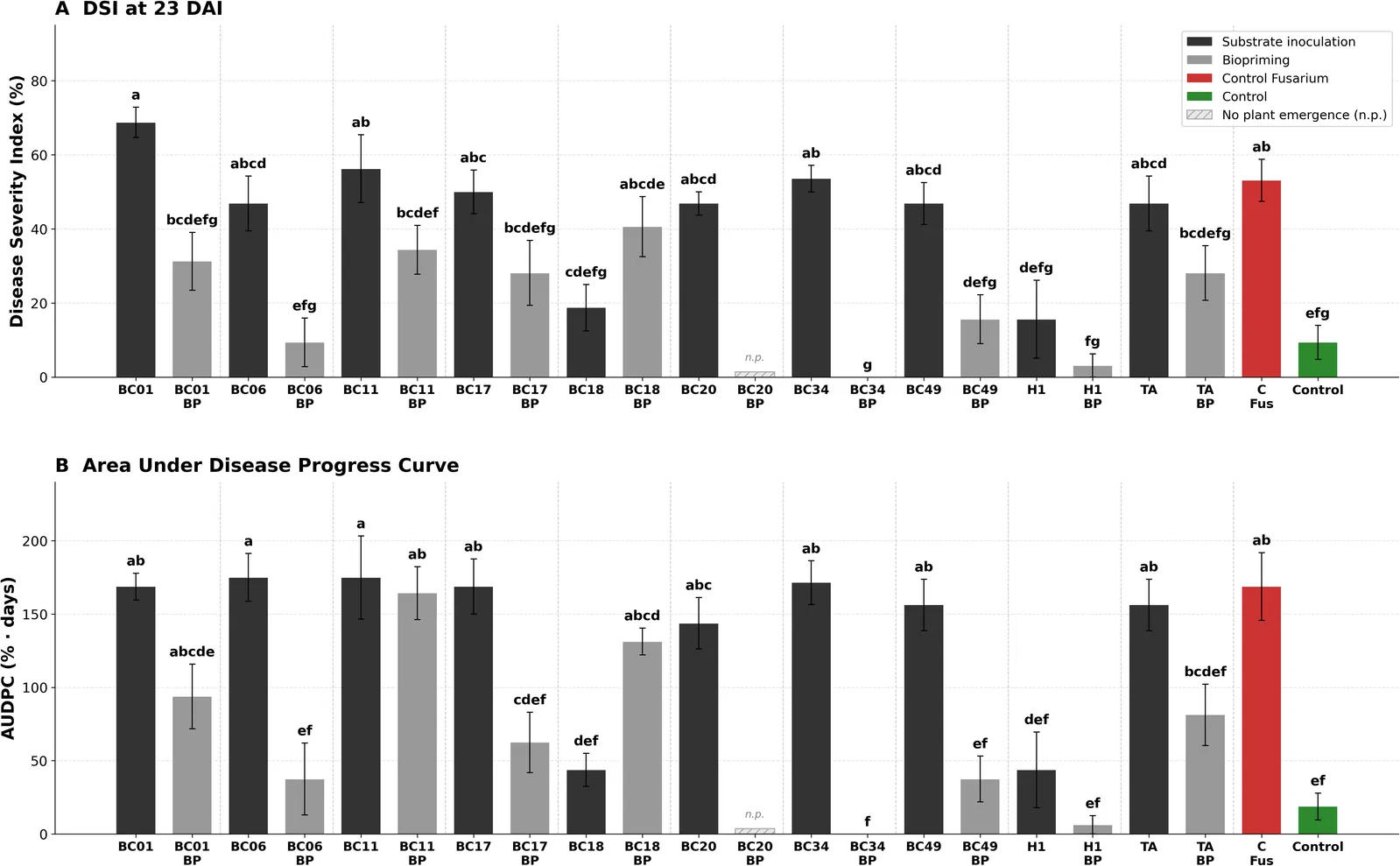
Disease severity index (DSI) at 23 days after inoculation (DAI) **(A)** and area under the disease progress curve (AUDPC) **(B)** for tomato plants treated with ten fungal isolates applied via substrate inoculation (dark grey bars) or seed biopriming (light grey bars), and inoculated with *Fusarium oxysporum* (f) sp. *lycopersici* (FOL). Control *Fusarium* (red) corresponds to FOL-inoculated plants without biocontrol treatment; Control (green) corresponds to non-inoculated, untreated plants. Values are means ± standard error (n = 10). Lowercase letters above bars indicate significant differences among treatments (Tukey’s HSD test, *p* < 0.05). BC20_BP is not shown: this treatment produced 0% seedling emergence (n = 30, three independent sowings), and disease variables were therefore not measurable.

The efficacy of the ten fungal isolates as biocontrol agents against FOL was evaluated by comparing two application methods, substrate inoculation and seed biopriming, in terms of disease severity index (DSI) and area under the disease progress curve (AUDPC) (**Figure 9**). Wilting and chlorosis incidence data recorded throughout the monitoring period are presented separately in **Supplementary Figures 1** and **S2**, respectively. Seed biopriming with *T. gamsii* (BC20) formulated in gum arabic (30% w/v) resulted in complete failure of seedling emergence: no seedling emerged from any of the 30 pots across three independent sowings. Because no plants were established, disease and growth variables could not be measured for this treatment; the emergence outcome itself is reported as a result of the isolate–matrix combination. BC20 applied by substrate inoculation established normally and conferred 11.8% protection, indicating that the effect is attributable to the biopriming formulation rather than to the isolate as such.

Disease severity at 23 days after inoculation (DAI) varied widely among treatments, ranging from 0% (BC34_BP) to 68.75% (BC01) among treatments with viable seedlings. Among substrate-inoculated treatments, BC01 showed the highest DSI, statistically comparable to the *Fusarium* control (53.13%), indicating that substrate application of this isolate failed to reduce disease severity relative to the untreated infected control. In contrast, biopriming with several isolates markedly reduced disease severity compared to their corresponding substrate-inoculated treatments, with relative DSI reductions of 54.5% for BC01, 80.0% for BC06, 38.9% for BC11, 43.8% for BC17, and 80.0% for H1. BC34_BP showed complete suppression of disease symptoms, with a DSI of 0% despite normal seedling emergence, representing the most effective treatment among all biocontrol combinations tested. BC06_BP (9.38%) and H1_BP (3.13%) also showed markedly low DSI values. An opposite trend was observed for BC18, where biopriming resulted in a higher DSI (40.63%) than substrate inoculation (18.75%), representing a 116.7% increase relative to its substrate-inoculated counterpart and suggesting an isolate-specific interaction between application method and disease outcome.

The AUDPC results paralleled the DSI patterns, confirming that biopriming reduced cumulative disease progression relative to substrate inoculation across most isolates. BC34_BP again showed the lowest AUDPC value (0), consistent with the complete absence of disease symptoms throughout the monitoring period, while H1_BP showed the second-lowest AUDPC among treatments with surviving seedlings (6.25). BC11_BP was a notable exception, showing an AUDPC (164.29) statistically similar to its substrate-inoculated counterpart, indicating limited protective benefit of biopriming for this particular isolate. As noted above, no AUDPC value could be calculated for BC20_BP. Overall, these results indicate that seed biopriming generally outperformed substrate inoculation in reducing both disease severity and cumulative disease progression, with BC34_BP achieving complete disease suppression. BC18 represented a notable exception to this general trend.

**Figure 10:**
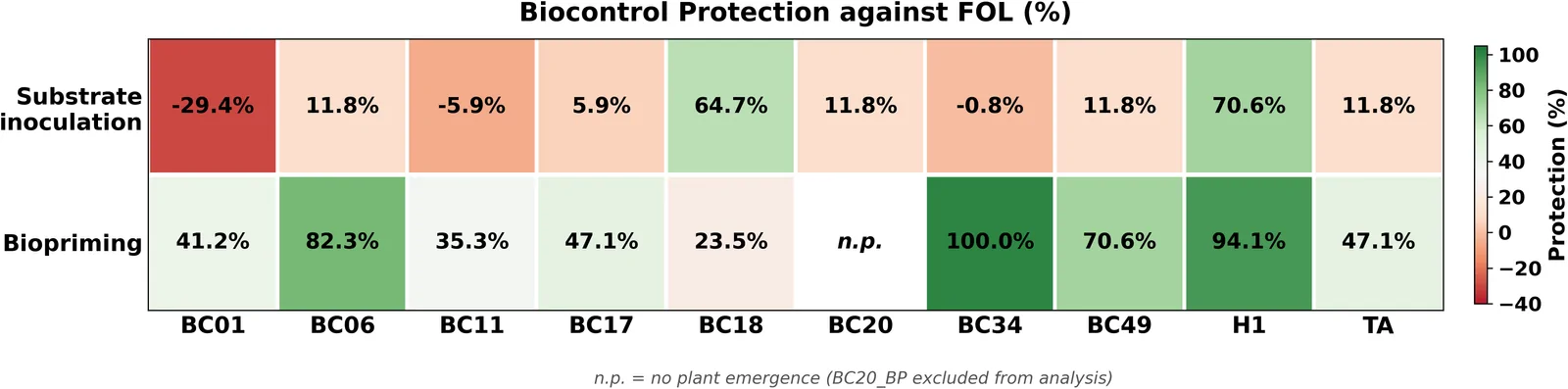
Biocontrol protection (%) against FOL conferred by ten fungal isolates applied via substrate inoculation (upper row) or seed biopriming (lower row), calculated relative to the Fusarium control. Cell color indicates the level of protection, ranging from red (negative protection, i.e., increased disease severity) through white (no effect) to green (high protection). Cell values represent the mean protection percentage. n.p., no plant emergence; BC20_BP: 0% seedling emergence (n = 30, three independent sowings); disease and growth variables not measurable.

**Figure 11:**
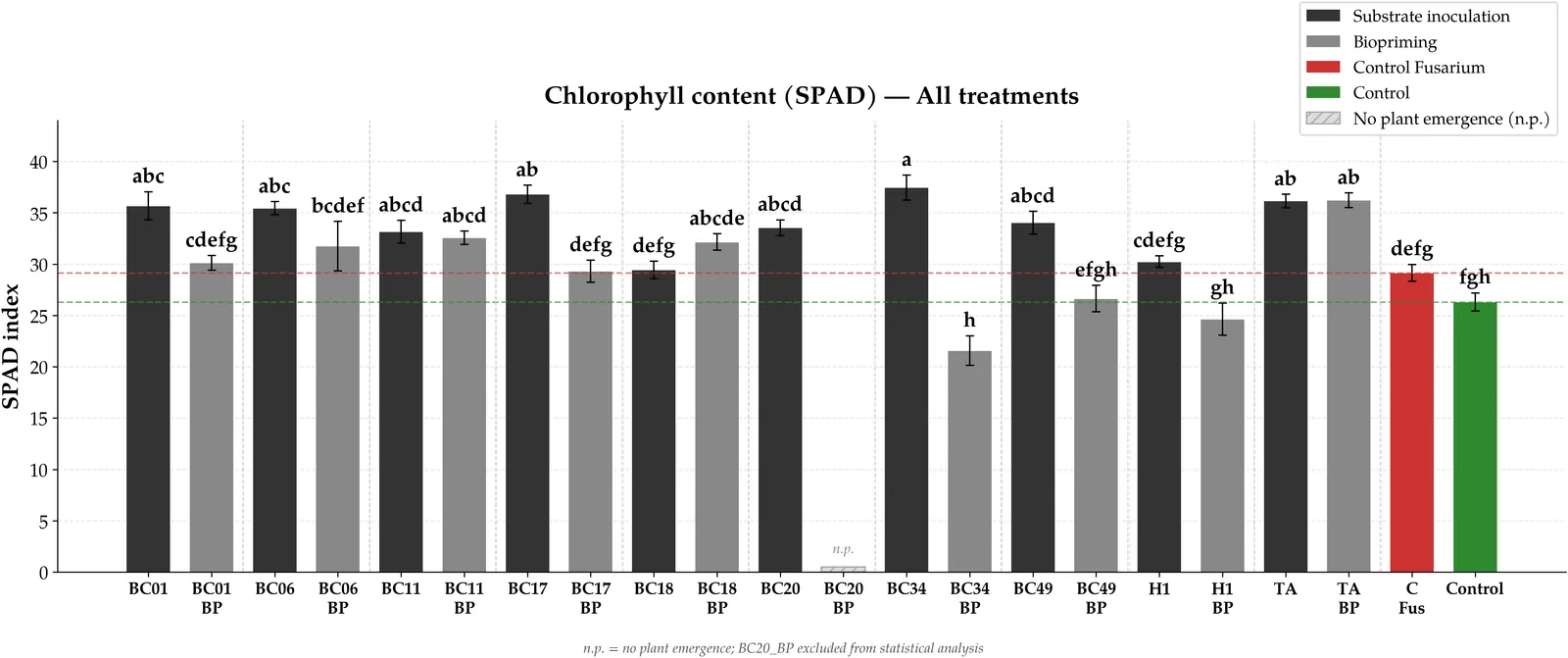
Chlorophyll content (SPAD index) at 23 days after inoculation (DAI) for tomato plants treated with ten fungal isolates applied via substrate inoculation (dark grey bars) or seed biopriming (light grey bars) and inoculated with FOL. Control Fusarium (red) corresponds to FOL-inoculated plants without biocontrol treatment; Control (green) corresponds to non-inoculated, untreated plants. The red and green dashed lines indicate the mean SPAD values of the Fusarium control and the non-inoculated control, respectively. Values are means ± standard error (n = 10). Lowercase letters above bars indicate significant differences among treatments (Tukey’s HSD test, *p* < 0.05). n.p., no plant emergence; BC20_BP, 0% seedling emergence (n = 30, three independent sowings); disease and growth variables not measurable.

To provide a comprehensive overview of biocontrol efficacy, the protection percentage conferred by each isolate–application method combination relative to the Fusarium control is shown in **Figure 10**. Overall, biopriming consistently outperformed substrate inoculation across most isolates, as evidenced by higher protection values in the lower row of the heatmap. BC34_BP achieved complete disease suppression (100% protection), representing the most effective biocontrol combination tested, followed by H1_BP (94.1%) and BC06_BP (82.4%). BC49_BP and H1 applied via substrate inoculation both reached 70.6% protection, confirming H1 as one of the most consistently effective isolates regardless of application method. In contrast, substrate inoculation with BC01 (-29.4%) and BC11 (-5.9%) resulted in negative protection values, indicating that these treatments exacerbated disease severity beyond that observed in the untreated Fusarium control. BC18 was the only isolate showing a reversed pattern, with higher protection under substrate inoculation (64.7%) than biopriming (23.5%), suggesting an isolate-specific incompatibility with the biopolymer formulation. Protection could not be assessed for BC20_BP (see above). Chlorophyll content, estimated through the SPAD index at 23 DAI, varied significantly across treatments (**Figure 11**). Substrate-inoculated treatments generally maintained higher SPAD values than their corresponding biopriming counterparts. BC34 showed the highest chlorophyll content among all treatments, followed by BC17 and both TA and TA_BP. In contrast, BC34_BP showed the lowest SPAD value across all treatments, representing a reduction of approximately 42% relative to its substrate-inoculated counterpart and the most pronounced decline observed among all biopriming treatments, despite achieving complete disease suppression. H1_BP and BC49_BP similarly showed reduced SPAD values compared to their respective substrate-inoculated treatments. Both control treatments, the Fusarium control and the non-inoculated control, fell within an intermediate range, statistically comparable to several biopriming treatments. Notably, TA was the only isolate for which biopriming did not reduce chlorophyll content relative to substrate inoculation, with both TA and TA_BP showing similarly high SPAD values (~36.2). No SPAD data were obtained for BC20_BP (see above). To further explore the relationship between application method and chlorophyll content, SPAD values obtained under substrate inoculation and biopriming were compared in a paired scatter plot (**Supplementary Figure 3**). The majority of isolates fell below the 1:1 diagonal, confirming that biopriming consistently reduced SPAD values relative to substrate inoculation in most cases. TA was the only exception, with both application methods yielding nearly identical SPAD values, suggesting that this isolate is particularly compatible with the biopolymer formulation in terms of maintaining plant photosynthetic capacity. BC18 was the only isolate where biopriming resulted in a slightly higher SPAD value than substrate inoculation, in contrast to its lower protection value under biopriming, reinforcing the complex and isolate-specific nature of the biopolymer–endophyte interaction. The Spearman correlation between substrate and biopriming SPAD values was not statistically significant (ρ: -0.23 p=0.546), indicating that the ranking of isolates by chlorophyll content differed substantially between the two application methods.

**Figure 12:**
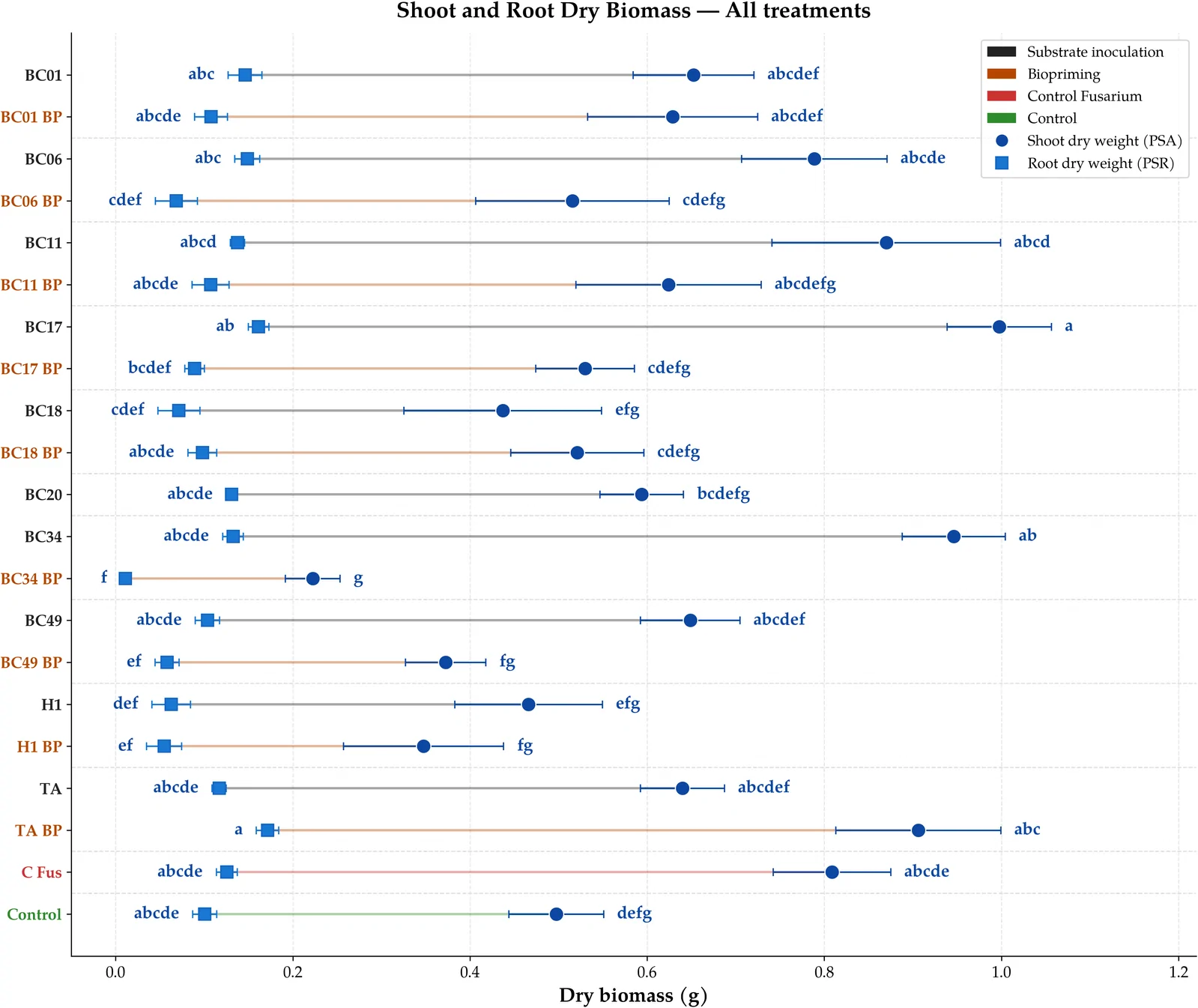
Shoot dry weight (PSA, circles) and root dry weight (PSR, squares) of tomato plants treated with ten fungal isolates applied via substrate inoculation (black lines) or seed biopriming (orange lines), and inoculated with FOL. Control Fusarium (red) corresponds to FOL-inoculated plants without biocontrol treatment; Control (green) corresponds to non-inoculated, untreated plants. Error bars indicate standard error of the mean (n = 30). Lowercase letters indicate significant differences among treatments for each variable separately (Tukey’s HSD test, p< 0.05).

Plant growth parameters measured at the end of the experiment are shown in **Figure 12** and **Supplementary Figures 4**, **S5**. Regarding shoot and root dry biomass (**Figure 12**), significant differences were detected among treatments. BC17 applied via substrate inoculation exhibited the highest shoot dry weight (PSA) of all treatments, followed by BC34 via substrate inoculation, both significantly higher than most biopriming treatments. In contrast, BC34_BP showed the lowest values for both PSA and PSR, consistent with the markedly reduced SPAD index observed for this treatment despite its complete disease suppression. TA_BP was notable for exhibiting the highest root dry weight (PSR) among all treatments, suggesting that biopriming with this isolate particularly promotes root dry weight accumulation. In general, biopriming tended to reduce shoot dry biomass relative to substrate inoculation across most isolates, with the most pronounced reductions observed for BC06_BP, BC49_BP, and H1_BP. However, TA_BP and BC18_BP did not follow this pattern, showing PSA values comparable to or higher than their substrate-inoculated counterparts. Stem diameter did not differ significantly among treatments, with all isolates and application methods yielding statistically comparable values (~0.4–0.5 cm). BC34 via substrate inoculation showed the greatest plant height, while BC34_BP again showed the lowest values, reinforcing the overall poor vegetative performance of this biopriming combination despite its outstanding biocontrol efficacy (**Supplementary Figures 4**, **S5**). The Fusarium control exhibited the highest leaf area among all treatments, which may reflect a compensatory growth response prior to the onset of severe disease symptoms rather than genuine growth promotion. Total dry biomass followed a similar pattern to shoot dry weight, with BC17 and BC34 via substrate inoculation showing the highest values and BC34_BP the lowest.

Overall, these results suggest that while biopriming with several isolates effectively suppressed FOL disease, this was frequently accompanied by a reduction in vegetative growth parameters relative to substrate inoculation, highlighting a potential trade-off between biocontrol efficacy and plant growth promotion depending on the isolate–application method combination.

**Figure 13:**
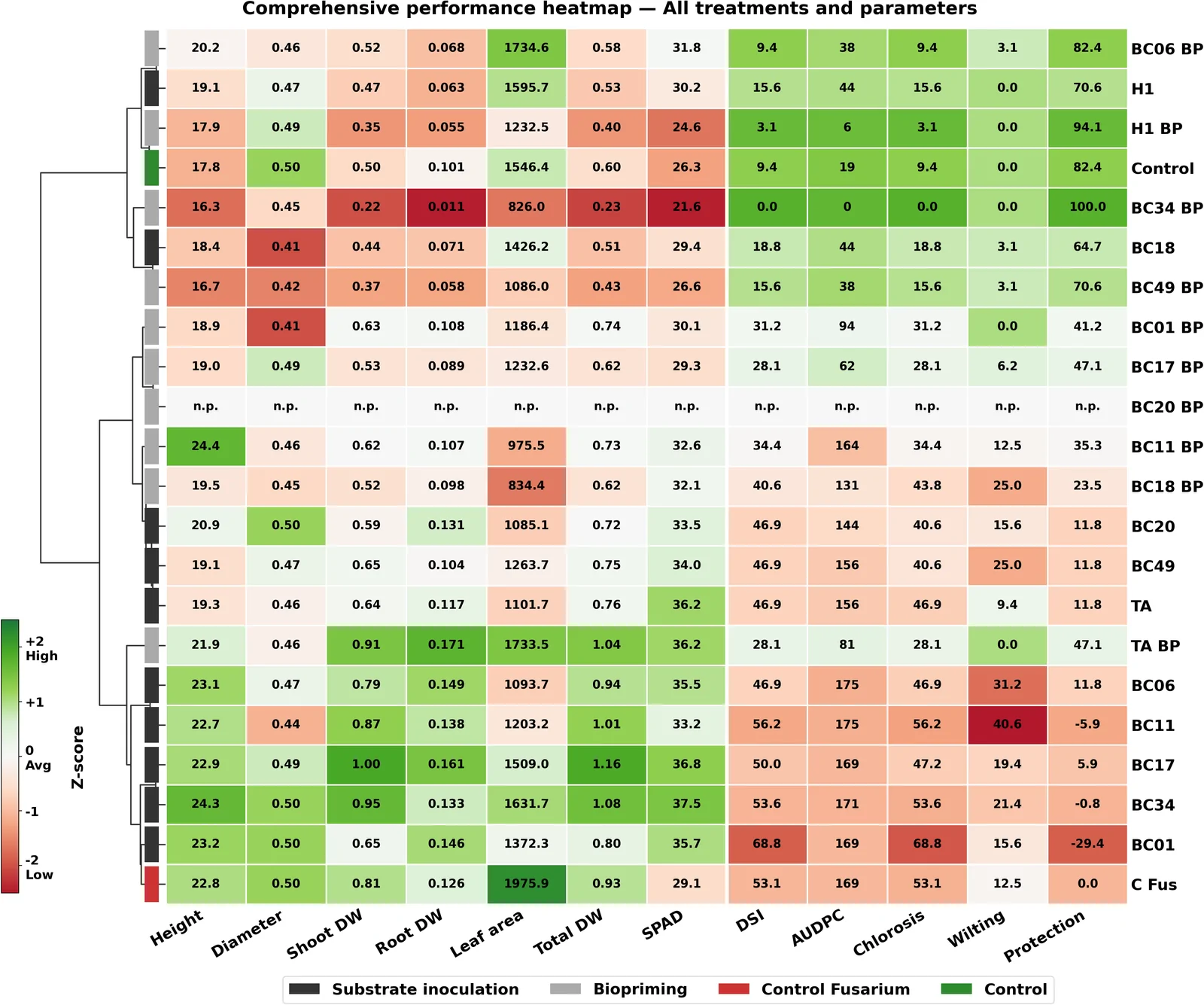
Comprehensive performance heatmap integrating growth and disease parameters across all biocontrol treatments. Rows represent fungal isolates ordered by Ward hierarchical clustering (dendrogram, left); columns are grouped by parameter type: growth variables (Height, Diameter, Shoot DW, Root DW, Leaf area, Total DW, SPAD) on the left, and disease-related variables (DSI, AUDPC, Chlorosis, Wilting, Protection) on the right, separated by a white vertical line. Cell values show original means; cell color represents the z-score normalized value, where green indicates above-average performance and red indicates below-average performance. For disease variables, the color scale is inverted so that green reflects better disease suppression (lower DSI, AUDPC, Chlorosis and Wilting; higher Protection). The colored strip to the left indicates treatment type. n.d., no data; n.p. = 0% seedling emergence (BC20_BP: 0% seedling emergence (n = 30, three independent sowings); disease and growth variables not measurable.).

The comprehensive heatmap integrating all growth and disease parameters across all treatments revealed two main clusters of isolates based on their overall performance profile (**Figure 13**). The upper cluster, dominated by biopriming treatments (BC34_BP, H1_BP, BC01_BP, BC18, BC49_BP), was characterized by lower growth parameter values (height, shoot and root dry weight, leaf area, total biomass) but markedly superior disease suppression, as reflected by low DSI, AUDPC, chlorosis and wilting values and high protection percentages. BC34_BP and H1_BP were the most prominent representatives of this cluster, achieving the highest protection values (100% and 94.1%, respectively) alongside the lowest SPAD index. The lower cluster, predominantly composed of substrate-inoculated treatments (BC17, BC34, BC11, BC06, BC01, TA_BP), showed the opposite pattern — higher growth parameters, particularly shoot dry weight and total biomass, but comparatively higher disease severity values. BC17 and BC34 via substrate inoculation stood out as the treatments with the highest shoot dry weight and total biomass, yet without a marked reduction in disease severity relative to the Fusarium control. TA_BP was a notable exception within this cluster, combining high shoot dry weight and root dry weight with intermediate disease suppression. The *Fusarium* control and several substrate-inoculated treatments with negative or near-zero protection values (BC01, BC11, BC34) clustered together in the lower right of the heatmap, reinforcing their limited biocontrol efficacy. Overall, the heatmap reveals a general trade-off between biocontrol efficacy and vegetative growth promotion, with biopriming treatments tending to suppress disease more effectively but at the cost of reduced plant biomass, while substrate-inoculated treatments generally supported greater plant growth but with less consistent disease control.

**Figure 14:**
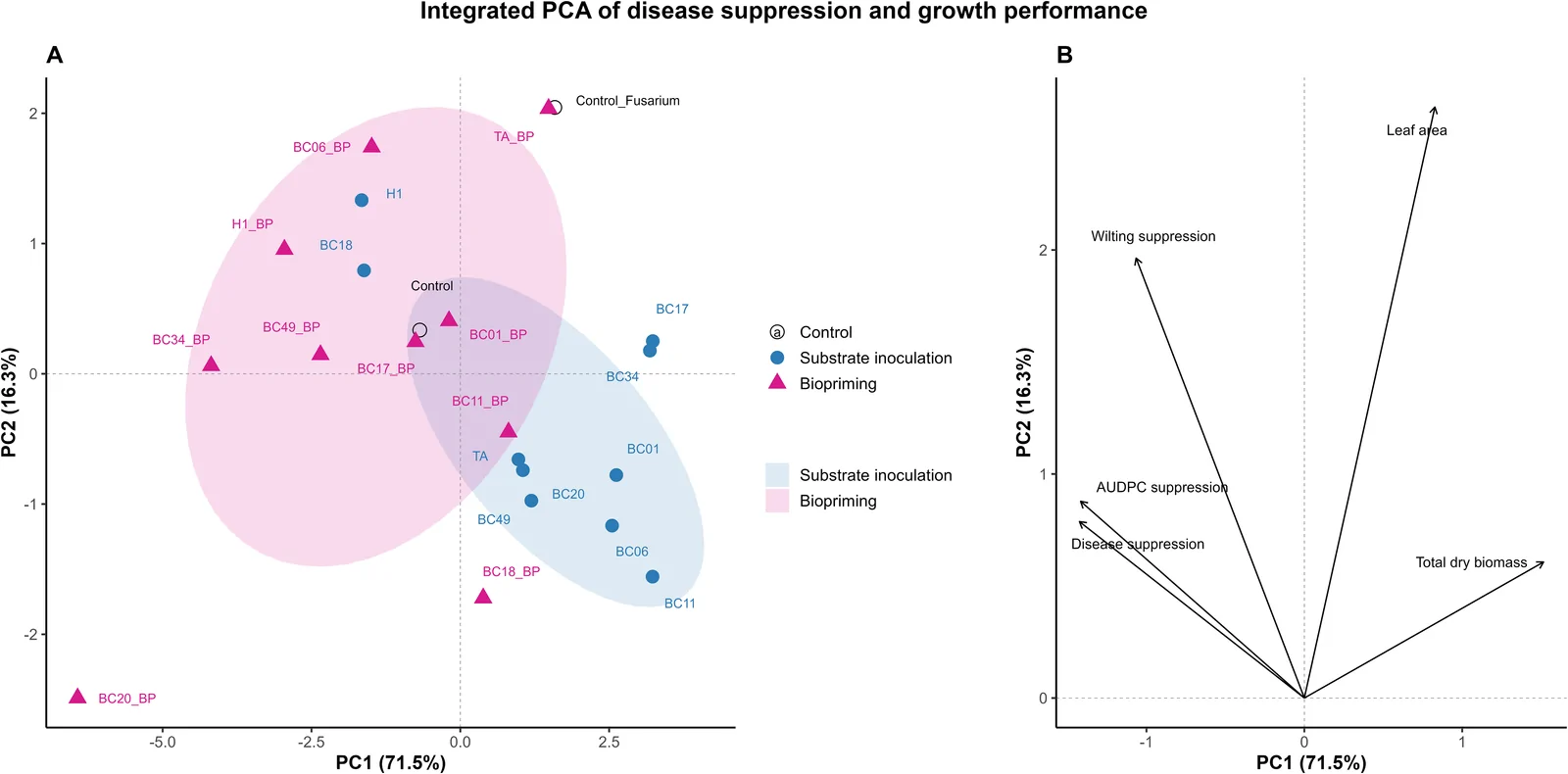
Principal component analysis (PCA) integrating disease suppression and growth performance variables across all biocontrol treatments. Panel **(A)** shows the score plot, where each point represents a treatment; circles indicate substrate inoculation and triangles indicate seed biopriming. The non-inoculated control is represented by the open circle symbol. Confidence ellipses (95%) are shown for substrate inoculation (blue) and biopriming (pink) treatment groups. Panel **(B)** shows the loading plot, illustrating the direction and magnitude of each variable’s contribution to PC1 and PC2. Variables pointing left (Disease suppression, AUDPC suppression, Wilting suppression) are associated with biopriming treatments; variables pointing right (Total dry biomass, Leaf area) are associated with substrate-inoculated treatments. The percentage of variance explained by each principal component is indicated on the corresponding axis. BC20_BP: 0% seedling emergence (n = 30, three independent sowings); disease and growth variables not measurable. Control_Fusarium = FOL-inoculated plants without biocontrol treatment.

To provide an integrated overview of biocontrol efficacy and plant growth performance across all treatments, a principal component analysis (PCA) was conducted incorporating disease suppression and growth variables (**Figure 14**, panel A). The first two principal components explained 87.8% of the total variance (PC1: 71.5%; PC2: 16.3%), indicating a strong underlying structure in the data. PC1 clearly separated biopriming treatments (negative scores, left side) from substrate-inoculated treatments (positive scores, right side), confirming that the application method was the primary driver of variation in the dataset. The loading plot (**Figure 14**, panel B) revealed that disease suppression variables (Disease suppression, AUDPC suppression, and Wilting suppression) loaded strongly and negatively on PC1, while growth-related variables (Total dry biomass and Leaf area) loaded positively. This pattern reinforces the trade-off between biocontrol efficacy and vegetative growth promotion observed in the individual parameter analyses. PC2 accounted for additional variation related to Wilting suppression and Leaf area, partially separating treatments within each application method group. The confidence ellipses for biopriming (pink) and substrate inoculation (blue) showed partial overlap in the central region of the score plot, indicating that some treatments from both methods share similar performance profiles. Notable outliers included BC20_BP, which was positioned far from all other biopriming treatments due to the absence of plant emergence and the consequent lack of representative data for most variables, and Control_Fusarium, which occupied an isolated position in the upper part of the plot consistent with its high disease severity and absence of biocontrol treatment. Taken together, the PCA confirms that biopriming and substrate inoculation represent two distinct performance strategies: biopriming favors disease suppression at the expense of vegetative growth, while substrate inoculation tends to support greater plant biomass with less consistent biocontrol efficacy.

## 4 Discussion

In the current context of sustainable agriculture, biopolymer-based seed coating has emerged as an innovative and environmentally friendly alternative to traditional chemical pesticides, enabling not only a reduction in environmental impact but also improved delivery efficiency of beneficial microorganisms to seeds (Zhang et al., 2025; Sprey et al., 2025; Xu et al., 2020; Cortés-Rojas et al., 2021). Biopolymers such as chitosan, alginate, cellulose, and pullulan are particularly notable for their biodegradability, low toxicity, and capacity to form protective matrices that preserve both seed viability and microbial agents during storage and following sowing (Zhang et al., 2025; Xu et al., 2020; Arpitha et al., 2024; Chin et al., 2022). This technology enables the precise incorporation of beneficial fungi such as *Trichoderma* spp. or *Pochonia chlamydosporia*, as well as plant growth-promoting bacteria, directly onto the seed surface through film coating or pelleting techniques (Usmanova et al., 2024; Sangeetha et al., 2025; Chin et al., 2022). Several studies have demonstrated that the use of biopolymers as carrier matrices promotes the viability and controlled release of microbial inoculants in the soil, thereby increasing germination rates, early seedling vigor, and tolerance to soilborne pathogens (Sprey et al., 2025; Malavathu et al., 2025; Usmanova et al., 2024; Xu et al., 2020; Chin et al., 2022). Furthermore, these coatings can be engineered to modulate physicochemical properties — such as water retention or adhesiveness — optimizing both the adhesion of the biological agent and its persistence under variable environmental conditions (Korbecka-Glinka et al., 2022; Chin et al., 2021; Rocha et al., 2019). Moreover, biopolymer coatings enriched with beneficial fungi have been reported not only to significantly reduce the incidence of diseases caused by phytopathogenic fungi such as *Fusarium* spp. or *Macrophomina* spp., but also to induce systemic defense responses in host plants and increase agricultural yields under different production systems (Sprey et al., 2025; Malavathu et al., 2025; Xu et al., 2020; Chien et al., 2022). Nevertheless, challenges remain regarding the standardization of formulations at commercial scale and their validation under diverse agroclimatic field conditions (Usmanova et al., 2024; Zhang et al., 2025).

The choice of biopolymer carrier not only conditions the physiological response of the seed but critically determines the viability and functionality of the encapsulated biological agent. Available evidence indicates that the choice of material and its concentration can substantially modify coating efficacy, as illustrated by the superior performance of 1.5% sodium alginate in retaining viable spores of *Trichoderma asperellum* without impairing germination, although increasing inoculant doses may reduce radicle elongation (Chin et al., 2021). This formulation-dependence is also observed in other systems, where polysaccharides such as pullulan, chitosan, or porous controlled-release matrices improve the physical stability of the coating, microbial survival, and protection against phytopathogens, with positive effects on germination, vigor, and early growth (Usmanova et al., 2024; Malavathu et al., 2025). However, outcomes are not uniform across crops, microorganisms, and environments, suggesting that the final effect of the coating depends on the interaction between biopolymer type, concentration, microorganism, and establishment conditions, rather than on the isolated contribution of any single factor (Cardarelli et al., 2022; Guevara-Fernandez et al., 2026; Rocha et al., 2019).

This potential is particularly well documented for *Trichoderma* spp., the genus concentrating the largest body of published evidence on biocontrol fungal formulations in biopolymer carriers. The combination of a biopolymer formulation with *T. harzianum* strain T22 in tomato increased total fruit yield and the accumulation of functional compounds such as lycopene and stress-related amino acids, indicating that the carrier itself can modulate the beneficial effect of the fungus on the crop (Carillo et al., 2020). Seed coating with *Trichoderma* conidia has also consistently improved germination, early growth, and systemic resistance against soilborne and foliar pathogens, including *Fusarium culmorum* in durum wheat (Kthiri et al., 2020) and downy mildew in cucumber (Szczech et al., 2017), confirming that the seed represents an effective vehicle for establishing the fungus in the root system. The incorporation of *T. harzianum* in alginate pellets, sometimes combined with osmotic agents such as PEG, has also been shown to promote hyphal proliferation in the soil after hydration, potentially translating into faster rhizosphere colonization (Knudsen et al., 1991). Although most of this evidence focuses specifically on *T. harzianum*, it provides a solid basis for extrapolating analogous formulations to species such as *T. gamsii*, *T. saturnisporum*, *T. longibrachiatum*, and *T. aggressivum*, even in the absence of strain-specific published data for all of them. Consistent with this body of work, gum arabic was the carrier that consistently sustained the highest and most reproducible germination across the four *Trichoderma* isolates evaluated in the present study (*T. gamsii* (BC20), *T. saturnisporum* (BC49), *T. longibrachiatum* (H1), and *T. aggressivum* f.sp. *europaeum* (TA)), reinforcing its suitability as a carrier for conidial inoculants of this genus.

Regarding dark septate endophytes (DSE), although most available studies have focused on their direct effects on plants under water or saline stress (Arriagada-Escamilla et al., 2025; Adeleke et al., 2024), emerging reports describe their formulation through microencapsulation for agricultural applications. Ye et al. (2025) optimized alginate microcapsules for two dominant DSE strains in wheat under drought conditions, obtaining significant improvements in biomass and yield parameters. Polymeric coatings have also been shown not only to protect the fungus but also to modulate rhizospheric interactions and promote more efficient colonization of the root system (Sagita et al., 2025). In this context, the results of the present study show that sodium alginate at low concentrations (0.5–1% w/v) was the most suitable carrier for microsclerotia-producing isolates (*Clarireedia calopus* (BC17, BC18) and *Clarireedia narcissi* (BC34)), while gum arabic proved detrimental for BC34 at most concentrations evaluated, revealing that compatibility between biopolymer and propagule is highly specific and cannot be generalized across isolates of the same functional group. These findings are consistent with the premise that the successful combined use of biopolymers and DSE depends both on rational matrix design and on fine-tuning the interaction between fungus, carrier, plant, and habitat (Newsham, 2011; Sagita et al., 2025), and underscore the need to evaluate compatibility on an individual basis before scaling any formulation to field conditions.

In the present study, biopolymer type, concentration, and imbibition time proved to be the primary determinants of seed germination and early seedling establishment, consistently overriding other sources of variation. Notably, imbibition at 30 min proved incompatible with germination regardless of biopolymer type or concentration evaluated, while 15 min emerged as the optimal exposure period, preserving germination above 80% across most treatments — a finding that underscores the critical role of imbibition kinetics in biopolymer-based seed coating systems. Sodium alginate (SA, 0.5–1% w/v) and gum arabic (GA, 25–40% w/v) consistently outperformed guar gum across germination and vigor parameters, suggesting that matrix viscosity, film-forming capacity, and porosity play a meaningful role in modulating the seed hydration microenvironment. These findings align with the premise that rational formulation design is not merely a technical refinement but a prerequisite for preserving seed physiological integrity — a consideration that has received comparatively little attention in the biopolymer and biocontrol literature, where compatibility with the microbial agent has frequently taken precedence over seed-side responses (Chin et al., 2022; Uthoff et al., 2024).

The reduction in germination observed under specific biopolymer–concentration combinations is unlikely to reflect a single inhibitory process. Rather, the evidence supports a multifactorial explanation in which oxygen limitation, osmotic restriction and mechanical restraint contribute to different extents depending on the film architecture generated by each matrix at each concentration (Corbineau, 2022; Corbineau et al., 2023). Restricted oxygen supply is the most parsimonious explanation for the losses associated with guar gum, which produced the most heterogeneous germination response and, given its viscosity-building capacity, is expected to impose the strongest diffusion barrier once hydrated. Oxygen is essential for the resumption of respiration during imbibition, and seed-covering layers can inhibit germination by limiting its delivery to the embryo (Corbineau, 2022). This is consistent with direct measurements in polymer-coated cereal seeds, where embryo oxygen concentrations fell by 90–99% relative to atmospheric levels and well below those of uncoated controls (Gorim and Asch, 2017), and with recent evidence that delayed germination in hydrogel-coated seeds is driven primarily by restricted gas exchange rather than by impaired water uptake, since exposing exchange sites such as the hilum and micropyle restores germination timing (Phillips et al., 2026). Aeration during priming likewise improves tomato germination by relieving oxygen limitation (Badek et al., 2005; Santika et al., 2022).

A second mechanism accounts for the decline recorded at the highest gum arabic concentrations, where the solute load at 25–40% w/v substantially lowers the water potential of the imbibition medium. Priming outcomes are highly sensitive to the water potential of the surrounding medium, and reduced water potential delays or suppresses radicle emergence even when preparatory metabolism proceeds normally (Corbineau et al., 2023; Dahal and Bradford, 1994). In tomato, osmotic stress can substitute quantitatively for mechanical restraint in inhibiting germination, indicating that reduced external water potential acts directly on the balance between embryo growth potential and the resistance of the surrounding tissues (Liptay and Schopfer, 1983). The concentration-dependent reduction in GP and SVI at the highest gum arabic concentrations is therefore consistent with an osmotic penalty imposed by the coating solution itself rather than with a toxic effect.

A third, non-exclusive explanation is mechanical restraint. Germination is completed only when the growth potential of the embryo exceeds the force opposed by the covering layers, and in tomato the principal barrier is the micropylar endosperm: gibberellins promote germination primarily by weakening this tissue rather than by acting on the embryo alone (Groot and Karssen, 1987; Haigh and Barlow, 1987), and localised weakening of the endosperm cell wall is a general prerequisite for radicle protrusion (Steinbrecher et al., 2025). A dense, continuous film such as that formed by gum arabic — visible by SEM as a cohesive organic matrix masking the cellular boundaries of the testa (**Figure 3D**) — may therefore act as an additional external barrier that less vigorous seeds fail to overcome, particularly where endosperm weakening remains incomplete. By contrast, sodium alginate at low concentrations produced reticular, porous, multilayer films that adapted to the seed surface microtopography (**Figures 3A–C**) and preserved germination and vigour at levels equivalent to or above the control. It should be noted, however, that recent work on hydrogel-coated seeds attributes delayed germination primarily to restricted gas exchange and explicitly rules out mechanical loading as the dominant factor in that system (Phillips et al., 2026), so the relative contribution of this mechanism in the present study remains to be established.

Beyond these matrix-dependent effects, the response to imbibition time itself cannot be attributed to a uniform hydration effect across treatments; rather, it appears to reflect the sensitivity of tomato seeds to the precise timing and distribution of water uptake during imbibition. In tomato, imbibition and embryo water content follow a triphasic dynamic, and radicle emergence appears to depend less on a simple increase in turgor than on the progressive relief of mechanical restriction imposed by surrounding tissues, particularly the endosperm (Haigh and Barlow, 1987). This interpretation is consistent with studies showing that priming improves germination by promoting water redistribution toward metabolically available fractions and advancing pre-germinative processes such as DNA replication, β-tubulin accumulation, and structural reorganization of the seed coat (Nagarajan et al., 2005; De Castro et al., 1995; Castro et al., 2000; Lechowska et al., 2019). Furthermore, the literature indicates that the timing and mode of water supply strongly modify imbibition kinetics and final germination performance, even when the optimal moisture content is similar, and that aeration during priming can accelerate germination by preventing oxygen limitations at critical phases (Badek et al., 2005; Santika et al., 2022). The germination response to imbibition time was non-monotonic, and notably it was not restricted to biopolymer-treated seeds: untreated control seeds imbibed for 30 min showed the same collapse in germination (2.33%) and the same recovery at 60 min (87.00%), indicating that the response reflects the hydration–dehydration regime rather than the carrier matrix. A plausible interpretation is that tolerance to the re-drying step of the coating protocol varies non-linearly with the degree of hydration attained. At 15 min, water uptake and metabolic reactivation remain limited and the seed is re-dried without appreciable damage; by 60 min, hydration has proceeded far enough to induce the protective and repair machinery characteristic of priming — antioxidant systems, LEA and heat-shock proteins, DNA repair and membrane reorganisation — which underlies the beneficial effects of controlled hydration (De Castro et al., 1995; Nagarajan et al., 2005; Lechowska et al., 2019). At the intermediate exposure, respiratory activity and ROS production may have resumed before these systems are fully expressed, leaving the seed transiently vulnerable to desiccation. This interpretation is consistent with evidence that desiccation tolerance in tomato is lost and re-established as a function of hydration state rather than of exposure time alone (Dahal and Bradford, 1994; Sano and Verdier, 2024; Rosyad et al., 2026). It should nonetheless be regarded as a hypothesis: it was not tested directly here, and it contrasts with Chin et al. (2021), who identified 0.5 h as the optimal imbibition time for sodium alginate in pak choy seed. Direct measurement of seed water content, electrolyte leakage and antioxidant activity across the imbibition curve would be required to establish it, and remains a priority for future work.

Encapsulation in biopolymers represents a robust strategy for overcoming a central limitation of biocontrol fungi: the rapid loss of viability and performance caused by prolonged storage and exposure to abiotic stress following application (Løvschall et al., 2024; Couceiro et al., 2025). For *Trichoderma* spp., this trend is particularly consistent: agar-based emulsions maintained approximately 100% viability at 3 months and over 70% at 6 months in *T. atrobrunneum* (Martínez et al., 2023); nanocellulose and carboxymethylcellulose matrices preserved approximately 10^8^ CFU mL^-^¹ after 1 year while free spores practically lost viability (Brondi et al., 2025); and alginate, chitosan, and nanocellulose formulations maintained 100% viability at 37 °C for 2 months in *T. longibrachiatum*, also preserving antagonistic capacity against *Fusarium oxysporum* (Arias-Chavarría et al., 2025). Beyond *Trichoderma*, analogous results have been reported in entomopathogenic fungi: in *Beauveria bassiana*, both spray drying and ionic gelation increased thermal and UV stability relative to unformulated material and maintained mortality rates of up to 90% (Felizatti et al., 2021); and cross-linked CMC beads maintained 85% germination after 5 months compared to 69% in non-encapsulated conidia (Zaldivar et al., 2026). Mechanistically, studies converge in attributing the protective effect of encapsulation to the creation of a buffered microenvironment that reduces desiccation and oxidative damage, and enables controlled release that favors persistence and establishment of the fungus (Felizatti et al., 2021; Zaldivar et al., 2026; Couceiro et al., 2025). However, the magnitude of this benefit is not uniform, as it depends on the encapsulating material, crosslinking agent, drying process, and fungal strain (Pinotti et al., 2026; Felizatti et al., 2021).

The stability of bioprimed seeds during cold storage depends critically on the interaction between biopolymer matrix, fungal strain, storage temperature, and duration — and no single factor in isolation explains the observed viability dynamics (Dolatabad, 2026; Santos-Díaz et al., 2022). Although the storage period evaluated here (30 days) is shorter than those typically assessed in the encapsulation literature (3–12 months), it reflects a practically relevant timeframe for commercial seed biopriming applications, where treated seeds are generally used within weeks of production. In the present study, cold storage at 4 °C for 30 days preserved viable fungal loads on most bioprimed seeds, with pronounced differences among isolates reflecting both isolate-specific biological traits and biopolymer–propagule compatibility. *Trichoderma saturnisporum* (BC49), *T. longibrachiatum* (H1), and *T. aggressivum* f.sp. *europaeum* (TA) showed the greatest viability retention, consistent with the known stability of *Trichoderma* conidia under refrigerated conditions and with the protective role of gum arabic as a film-forming carrier. In contrast, *Sordaria fimicola* (BC06), *Trichoderma gamsii* (BC20), and *Clarireedia narcissi* (BC34) showed the most pronounced viability declines, reaching undetectable or near-zero loads by day 30. Sodium alginate at low concentrations (0.5–1% w/v) effectively preserved the viability of microsclerotia-producing isolates during storage, consistent with the literature showing that 4% sodium alginate maintained higher viability than 6% alginate or unencapsulated seeds in *Aquilaria malaccensis* after 30 days at both 4 °C and 25 °C (Kharnaior and Thomas, 2024), and that alginate-encapsulated conidia of *Beauveria bassiana* and *Metarhizium anisopliae* remained stable for 12 months compared to a drop to 46% viability in unencapsulated conidia by month six (Rodrigues et al., 2017). Gum arabic consistently sustained the highest viability across *Trichoderma* isolates, consistent with its known film-forming properties and moisture-buffering capacity, while guar gum showed the most variable performance and the lowest germination and vigor values across treatments, suggesting it requires further optimization before it can be recommended as a carrier for endophytic fungal biopriming in tomato. Notably, initial propagule entrapment efficiency and viable load at 30 days were not significantly correlated (rho = 0.43, p = 0.218), and isolates with comparable entrapment differed markedly in persistence, indicating that higher initial loading does not guarantee long-term persistence and that intrinsic biological traits of each isolate play a decisive role in survival beyond the physical retention capacity of the matrix. As noted by Santos-Díaz et al. (2022), standardized methods for assessing viability, release, and particle size in encapsulated biocontrol fungi are still lacking, which limits direct comparison across studies and highlights the need for system-specific optimization.

The biocontrol efficacy of the ten endophytic fungal isolates against *Fusarium oxysporum* f. sp. *lycopersici* (FOL) was evaluated by comparing two application methods — seed biopriming and substrate inoculation — in terms of disease severity index (DSI), area under the disease progress curve (AUDPC), and biocontrol protection percentage. The results demonstrated that seed biopriming consistently outperformed substrate inoculation across most isolate–biopolymer combinations, with *Clarireedia narcissi* (BC34_BP) and *Trichoderma longibrachiatum* (H1_BP) achieving complete or near-complete disease suppression (100% and 94.1% protection, respectively). These results are particularly noteworthy given that *C. narcissi* and, more broadly, dark septate endophytes as a group have not previously been evaluated as biocontrol agents against FOL in tomato, representing a relevant contribution to the understanding of the biological control potential of this largely overlooked fungal group in the tomato–*Fusarium* pathosystem.

The superior performance of biopriming over substrate inoculation observed in the present study is consistent with the hypothesis that direct seed coating places the biocontrol agent in immediate proximity to the emerging radicle and the soil–root interface at the most critical moment for pathogen exclusion (Rocha et al., 2019; Fan et al., 2024). Encapsulation in biopolymers further enhances this advantage by improving propagule persistence, controlling inoculant release, and protecting the fungus from abiotic stresses during pre-germination and early colonization (Riseh et al., 2022; Fan et al., 2024). This mechanistic advantage may explain why several isolates that showed limited protection under substrate inoculation — including *Torula* sp. (BC01), *Clarireedia calopus* (BC17), and *Trichoderma saturnisporum* (BC49) — achieved markedly higher protection values when applied as bioprimed seeds, suggesting that proximity and timing of inoculant delivery are as important as the intrinsic antagonistic capacity of the isolate.

Among the *Trichoderma* isolates, *T. longibrachiatum* (H1) showed the highest and most consistent biocontrol efficacy, with 94.1% protection under biopriming and 70.6% under substrate inoculation — the only isolate achieving high protection under both application methods. This is consistent with the broad biocontrol spectrum reported for *Trichoderma* spp. against *Fusarium* in tomato and other crops (Kthiri et al., 2020; Huertas et al., 2026), including competition for nutrients and space, mycoparasitism, and induction of systemic resistance. In contrast, *T. gamsii* (BC20) showed moderate protection under substrate inoculation but produced no seedling emergence under biopriming across three independent sowings. Notably, this outcome cannot be attributed to a failure of the formulation itself: BC20 in gum arabic (30% w/v) achieved 62.0% propagule entrapment and the highest absolute spore germination recorded in the compatibility assays (~9 × 10^5^ spores mL^-^¹; **Figures 1**, **2**). The coating therefore performed well from the standpoint of the bioagent while proving incompatible with the crop, which points to the delivered fungal load rather than the matrix as the proximate cause. Consistent with this, high conidial concentrations of *Trichoderma* spp. have been reported to inhibit radicle elongation in vegetable seeds, an effect attributed to the overproduction of metabolites such as harzianolide and peptaibols (Chin et al., 2021), although phytotoxicity of the formulation and oxygen restriction beneath the film during imbibition cannot be excluded with the present data. The complete disease suppression achieved by *C. narcissi* (BC34_BP) is a particularly relevant finding, as *Clarireedia* spp. are microsclerotia-forming dark septate endophytes with no previously documented biocontrol activity against FOL in tomato. The mechanisms by which BC34_BP achieved 100% protection remain to be elucidated, but may include competition for infection sites at the root surface, production of secondary metabolites with antifungal activity, and induction of systemic defense responses — mechanisms previously described for DSE in other crop–pathogen systems (Santos et al., 2021; Mariani et al., 2024). Interestingly, this complete suppression was accompanied by a marked reduction in plant biomass and SPAD index. Several non-exclusive explanations may account for this, which are examined in the following section.

*Clarireedia calopus* (BC18) was a notable exception to the general pattern, showing higher protection under substrate inoculation (64.7%) than under biopriming (23.5%). This reversed pattern suggests an isolate-specific incompatibility between BC18 and the biopolymer matrix under biopriming conditions, possibly related to the sensitivity of this isolate’s microsclerotia to the hydration dynamics imposed by the coating during imbibition, or to a differential capacity for rhizosphere establishment depending on the delivery route. A comparable isolate-specific reversal in biocontrol efficacy depending on the application method has been reported in other endophyte systems, reinforcing the need for individual evaluation of isolate–carrier–method combinations before recommending any formulation for practical use (Cardarelli et al., 2022; Rocha et al., 2019).

In contrast to BC18, substrate inoculation with *Torula* sp. (BC01 and BC11) resulted in negative protection values (-29.4% and -5.9%, respectively), indicating that these treatments exacerbated disease severity beyond that of the untreated Fusarium control. This counterintuitive outcome may reflect competition between these isolates and the plant’s own defense system, a disruption of the microbial community in the rhizosphere, or even a facilitative interaction between these fungi and FOL under substrate colonization conditions. In seed biopriming, however, both isolates showed positive protection values (41.2% and 35.3%, respectively), suggesting that the biopriming format partially overcame this negative effect — possibly by restricting the ecological window in which the adverse interaction could develop.

The reduction in chlorophyll content (SPAD index) and vegetative growth parameters observed in several biopriming treatments — most notably BC34_BP — despite effective or complete disease suppression, reflects a well-documented biological phenomenon known as the growth–defense trade-off (Gao et al., 2024; He et al., 2022; Figueroa-Macías et al., 2021). In the present study, BC34_BP achieved 100% protection against FOL while simultaneously showing the lowest SPAD index (21.57) and the lowest shoot and root dry weight among all treatments, representing a reduction of approximately 42% in chlorophyll content relative to its substrate-inoculated counterpart. This pattern is biologically consistent with a scenario in which complete disease suppression is sustained by a prolonged and resource-costly activation of plant immune responses, rather than by efficient and transient biocontrol mechanisms.

The growth–defense trade-off is well established in plant biology and crop science. High resistance activation is frequently associated with penalties in growth and yield, as immune responses divert carbon, nitrogen, and energy from primary metabolism toward the synthesis of defensive compounds (Gao et al., 2024; Karasov et al., 2017). Two main mechanisms have been proposed: a passive reallocation hypothesis, in which resources are physically diverted from growth toward defense; and an active regulatory hypothesis, in which plant hormonal networks — including salicylic acid (SA), jasmonic acid (JA), and the TOR kinase pathway — actively redirect metabolic flux (Ku et al., 2024; Chen, 2025). Systemic acquired resistance (SAR), in particular, has been shown to suppress photosynthesis and cause growth penalties of 5–15% in cereals (Chen, 2025), while reactive oxygen species (ROS), phenolic compounds, and defensive enzymes, when chronically elevated, can be associated with reduced growth even in the absence of active pathogen damage (Ayaz et al., 2024; Figueroa-Macías et al., 2021). Importantly, biomass reduction can occur without physical tissue loss, driven purely by systemic defense signaling (He et al., 2022), which is consistent with the morphologically intact but reduced plants observed in BC34_BP treatments in the present study.

However, the growth–defense trade-off is not an inevitable outcome of effective biocontrol. In tomato, seed coating with *Bacillus aryabhattai* Z-48 reduced disease index by more than 60% while simultaneously increasing root length, dry biomass, and total chlorophyll (Akram et al., 2024), demonstrating that biocontrol can be achieved without penalizing plant growth when the biological agent combines direct antagonism with transient and well-timed induction of plant defenses. Similarly, *Trichoderma virens* DM5 showed a biocontrol-biostimulant trade-off in tomato that depended on the intensity and duration of the immune response triggered, with lower-level induction being compatible with growth promotion (Thaiparambil et al., 2025). These examples suggest that the scenario of complete disease suppression combined with reduced biomass and SPAD — as observed for BC34_BP in the present study — arises specifically when defense activation is sustained and resource-costly, rather than transient and synergistic with growth. Although the growth–defense trade-off offers a plausible framework for interpreting the BC34_BP phenotype, the present results do not allow this mechanism to be considered exclusive, since endophyte-mediated effects on plant performance are context dependent and vary with host genotype, microbial identity, environmental conditions and the balance between colonization and host regulation (He et al., 2022; Mengistu, 2020; Bard et al., 2024). A first non-exclusive alternative is that the coating matrix itself, or metabolites released during seed imbibition, exerted a direct phytotoxic effect, since seed-applied formulations can inhibit germination or early growth depending on their composition and the stress context, even when designed for protection (Zhang et al., 2020; Amoroso et al., 2025). A second possibility is that the biomass penalty reflected the physiological cost of endophytic establishment, as fungal endophytes rely on host resources and their net effect shifts with nutrient exchange and colonization intensity (Waqar et al., 2023; Bard et al., 2024). A third explanation is that the high propagule load delivered by seed biopriming promoted a displacement along the mutualism–parasitism continuum, since endophytes can alter their lifestyle across plant developmental stages and under adverse conditions, and maintaining a beneficial association requires tight regulation of fungal proliferation and of endophyte-derived toxic activities (Lu et al., 2021; Liao et al., 2025). Discriminating among these alternatives will require direct quantification of root colonization by qPCR or reisolation — seed-applied fungi do not always establish true endophytism and may persist only epiphytically (Baron and Rigobelo, 2021) — together with expression analysis of defense-related genes, histological assessment of root tissue integrity, and a biopolymer-only coating control lacking fungal inoculum. The interpretation offered above should therefore be regarded as a hypothesis consistent with the observed phenotype rather than as a demonstrated mechanism.

In contrast, TA_BP (*T. aggressivum* f.sp. *europaeum*) was the only biopriming treatment that did not reduce SPAD relative to substrate inoculation, with both TA and TA_BP showing similarly high SPAD values (~36.2). This suggests that *T. aggressivum* is capable of inducing effective protection without imposing a significant physiological cost on the plant — a pattern consistent with microbial mechanisms that shift from trade-off to synergy by simultaneously promoting plant growth and immunity (Ku et al., 2024). The high root dry weight observed for TA_BP, the highest among all biopriming treatments, further supports this interpretation, indicating that this isolate may combine biocontrol activity with root growth promotion under the conditions evaluated.

The comprehensive heatmap and PCA integrating all parameters confirmed the existence of two distinct performance clusters: one dominated by biopriming treatments associated with high disease suppression but reduced growth and chlorophyll content, and another dominated by substrate-inoculated treatments associated with higher vegetative biomass but lower biocontrol efficacy. This trade-off pattern was particularly evident along PC1, which explained 71.5% of the total variance and clearly separated biopriming from substrate inoculation based on their contrasting profiles of disease suppression versus growth promotion. These results highlight that the selection of a biopriming formulation for practical use should not be based solely on disease suppression efficacy, but should consider the full agronomic performance profile of each isolate–biopolymer combination, including its effect on plant biomass, chlorophyll content, and seedling establishment.

## 5 Conclusion

This study demonstrates that biopolymer-based seed biopriming represents a viable and effective strategy for the delivery of endophytic fungal biocontrol agents against *Fusarium oxysporum* f. sp. *lycopersici* (FOL) in tomato, with performance outcomes that were consistently superior to substrate inoculation across most isolate–biopolymer combinations evaluated. To our knowledge, this is the first study to evaluate dark septate endophytes (DSE) — including *Clarireedia calopus*, *Clarireedia narcissi* — together with other understudied endophytic ascomycetes such as *Sordaria* fimicola, as biocontrol agents against FOL in tomato, and the first to demonstrate the feasibility of encapsulating DSE propagules in biopolymer matrices for seed biopriming applications. These findings open a new research avenue for an understudied fungal group with significant biotechnological potential.

Imbibition time was identified as the critical determinant of seed–biopolymer compatibility, with 15 min emerging as the optimal exposure period. Sodium alginate (SA, 0.5% w/v) and gum arabic (GA, 25–35% w/v) provided the most consistent germination and vigor performance, while guar gum showed higher variability. Propagule type strongly influenced biopolymer compatibility: spore-producing isolates achieved maximum viability with gum arabic at medium-to-high concentrations, whereas microsclerotia-producing isolates — the DSE *Clarireedia* spp. — were better preserved by sodium alginate at low concentrations, providing the first experimental evidence for the compatibility of this biopolymer with DSE propagules. Sodium alginate at 0.5% w/v was likewise the optimal carrier for the ascospore-producing isolate *Sordaria fimicola* (BC06), the only spore-forming isolate for which alginate outperformed gum arabic. Cold storage at 4 °C for up to 30 days preserved viable fungal loads on bioprimed seeds, with *T. saturnisporum* (BC49), *T. longibrachiatum* (H1), and *T. aggressivum* f.sp. *europaeum* (TA) showing the greatest persistence. Initial propagule entrapment efficiency and long-term viability were not significantly correlated, and isolates with comparable entrapment differed markedly in persistence, indicating that intrinsic biological traits of each isolate play a decisive role in survival beyond the physical loading capacity of the matrix. Germination performance of bioprimed seeds remained stable throughout 40 days of storage, confirming that the biopolymer–fungal formulations did not compromise seed physiological quality.

In the biocontrol assays, seed biopriming consistently outperformed substrate inoculation in reducing disease severity. Among the DSE isolates, *Clarireedia narcissi* (BC34_BP) achieved complete disease suppression (100% protection), representing the most effective biocontrol combination in the entire experiment and the first documented evidence of biocontrol activity of this species against FOL in tomato. *T. longibrachiatum* (H1_BP) achieved 94.1% protection, confirming its broad-spectrum antagonistic potential. The PCA and comprehensive heatmap revealed a clear trade-off between biocontrol efficacy and vegetative growth promotion, with biopriming treatments clustering towards greater disease suppression but reduced plant biomass, while substrate-inoculated treatments generally supported higher vegetative growth with less consistent disease control. *Clarireedia calopus* (BC18) was a notable exception, showing a reversed pattern with higher protection under substrate inoculation than biopriming, suggesting isolate-specific incompatibility with the biopolymer matrix. Notably, BC34_BP showed complete disease suppression despite a marked reduction in SPAD and growth parameters, the basis of which could not be established with the present data.

Overall, the results highlight the importance of individually optimizing biopolymer formulations for each fungal taxon and confirm that seed biopriming with biopolymer-encapsulated endophytic fungi — including previously undescribed DSE biocontrol agents — is a promising, scalable, and seed-compatible strategy for sustainable *Fusarium* wilt management in tomato. Future research should prioritize the elucidation of the mechanisms underlying DSE-mediated disease suppression, the optimization of formulations to mitigate the growth–defense trade-off, and the validation of the most effective isolate–biopolymer combinations under field conditions.

## Data Availability

The original contributions presented in the study are included in the article/**Supplementary Material**, further inquiries can be directed to the corresponding author/s.
