# Supplementary material for: Biopolymer-based seed biopriming with fungal endophytes suppresses Fusarium wilt and modulates growth in tomato
Source: Front Plant Sci. 2026 Sep 11;17:1931116. doi: 10.3389/fpls.2026.1931116 (PMC13612569; doi:10.3389/fpls.2026.1931116)
Supplement: Supplementary file 1 [file SupplementaryFile1.docx]

Supplementary Material

## 1.1 Supplementary Figures

**Supplementary Figure S1**. Wilting incidence (%) in tomato plants inoculated with fungal endophytes against FOL at 19 and 23 days after inoculation (DAI). Bars represent the mean ± SE (n = 30). Treatments include substrate inoculation (blue bars, Day 19; grey bars, Day 23) and seed biopriming (BP; light blue bars, Day 19; light grey bars, Day 23). Control Fusarium: plants inoculated with FOL without biocontrol agent. Control: healthy uninoculated plants. Different letters above bars indicate significant differences between treatments within each time point (Tukey's HSD test, p < 0.05).

**Supplementary Figure S2**. Chlorosis incidence (%) in tomato plants inoculated with fungal endophytes against FOL at 19 and 23 days after inoculation (DAI). Bars represent the mean ± SE (n = 30). Treatments include substrate inoculation (blue bars, Day 19; grey bars, Day 23) and seed biopriming (_BP; light blue bars, Day 19; light grey bars, Day 23). Control Fusarium: plants inoculated with FOL without biocontrol agent. Control: healthy uninoculated plants. Different letters above bars indicate significant differences between treatments within each time point (Tukey's HSD test, p < 0.05).

**Supplementary Figure S3.** Relationship between SPAD index values obtained under substrate inoculation and seed biopriming for each fungal isolate at 23 days after inoculation (DAI). Each point represents one isolate with data for both application methods. Error bars indicate standard error of the mean. The dashed diagonal line represents the 1:1 reference line (no difference between methods); points above the line indicate higher SPAD under biopriming, and points below indicate lower SPAD under biopriming. The shaded green and red areas indicate zones where biopriming improves or reduces chlorophyll content relative to substrate inoculation, respectively. Dotted lines indicate the mean SPAD of the Fusarium control. Spearman rank correlation coefficient (ρ) and associated p-value are shown. BC20_BP is excluded due to the absence of seedling emergence.

**Supplementary Figure S4**. Stem diameter (A) and total dry biomass (B) of tomato plants treated with ten fungal isolates applied via substrate inoculation (dark grey bars) or seed biopriming (light grey bars) and inoculated with FOL. Control *Fusarium* (red) and Control (green) are shown for reference. Individual data points (open circles) represent replicate values. Error bars indicate standard error of the mean (n = 10). Lowercase letters indicate significant differences among treatments (Tukey's HSD test, p < 0.05).

**Supplementary Figure S5**. Plant height (A) and leaf area (B) of tomato plants treated with ten fungal isolates applied via substrate inoculation (dark grey bars) or seed biopriming (light grey bars) and inoculated with FOL. Control Fusarium (red) and Control (green) are shown for reference. Individual data points (open circles) represent replicate values. Error bars indicate standard error of the mean (n = 10). Lowercase letters indicate significant differences among treatments (Tukey's HSD test, p < 0.05).
